# Potential Molecular Biomarkers of Vestibular Schwannoma Growth: Progress and Prospects

**DOI:** 10.3389/fonc.2021.731441

**Published:** 2021-09-27

**Authors:** Yu Zhang, Jianfei Long, Junwei Ren, Xiang Huang, Ping Zhong, Bin Wang

**Affiliations:** ^1^ Department of Pharmacy, Huashan Hospital, Fudan University, Shanghai, China; ^2^ Department of Neurosurgery, Huashan Hospital, Fudan University, Shanghai, China

**Keywords:** vestibular schwannoma, growth, biomarker, target therapy, merlin

## Abstract

Vestibular schwannomas (VSs, also known as acoustic neuromas) are relatively rare benign brain tumors stem from the Schwann cells of the eighth cranial nerve. Tumor growth is the paramount factor for neurosurgeons to decide whether to choose aggressive treatment approach or careful follow-up with regular magnetic resonance imaging (MRI), as surgery and radiation can introduce significant trauma and affect neurological function, while tumor enlargement during long-term follow-up will compress the adjacent nerves and tissues, causing progressive hearing loss, tinnitus and vertigo. Recently, with the deepening research of VS biology, some proteins that regulate merlin conformation changes, inflammatory cytokines, miRNAs, tissue proteins and cerebrospinal fluid (CSF) components have been proposed to be closely related to tumor volume increase. In this review, we discuss advances in the study of biomarkers that associated with VS growth, providing a reference for exploring the growth course of VS and determining the optimal treatment strategy for each patient.

## 1 Introduction

VSs are histologically benign lesions deriving from the Schwan cells of the vestibulo-cochlear nerve commonly occur unilaterally, or bilaterally in the pathognomonic for the hereditary disorder neurofibromatosis type 2 (NF2) (1, 2). Generally, the mere presence of benign tumors is not a therapeutic indication. However, the slow but progressive growth of this intracranial tumor can confer compression to adjacent cranial nerve and brainstem, resulting in neurological deficits, balance difficulty and sensorineural hearing loss (SNHL) (3).

The goal of VS treatment has shifted from saving lives towards functional preservation, with multifaceted decision including watch-wait-rescan protocol, surgical resection and radiation (4, 5). However, surgery can be traumatic (6), while radiotherapy has a low hearing retention rate and can affect neurological function even after many years (7). We can see from the literature that two-thirds of VS did not grow during 3.6 years of follow-up, the average growth rate of sporadic VS was 1.1 mm/year diameter, and for NF2-related tumors 1.7 mm/year (8, 9), supporting the wait and watch policy in appropriate patients. A study showed that conservative management in small‐ to medium‐sized VS (less than 2cm) can improve rates of facial nerve preservation and hearing protection in comparison to patients who undergo primary surgical treatment (10). Therefore, for stable or involuting tumors with no mass effect, or elderly patients who will suffer higher risk of comorbidities, the continuing trend toward observation with regular follow-up imaging is reasonable (5, 11).

Nevertheless, observation policy carries the inherent risk of tumor progression and hearing deterioration in growing tumors, which emphasizing the importance of the identification of effective and convenient indicators to predict growth characteristics at the time of diagnosis to weigh the benefits and risks of active treatment against watchful observation. Previous studies have reported onset at an early age, increasing size in the first year, hearing loss, extrameatal tumor location, cerebellopontine angle extension, sway velocity with eyes open were risk factors for tumor growth (9, 12–15). Here, we provide a narrative review on the topic of biomarkers currently considered to be related to VS growth in order to assist surgeons to select the most accurate treatment approach and the development of innovative targeted therapies.

## 2 Methods

The authors conducted a literature review using Web of Science and PubMed databases in order to identify important recent publications and synthesized them into a comprehensive review of potential biomarkers of VS growth. The search strategy included the search terms “growth”, “expression” and “vestibular schwannoma”. We have also scanned the reference lists of published articles for further potential hits of relevance to this review. Peer-reviewed full articles published in English till August 2021 were included to compile this review. Search results were judged for relevance by the research team using the title, abstract and if necessary full text. Studies were included, if they 1) explored the relationship between biomarkers and the size or growth of sporadic VS or NF2-associated VS; 2) explored molecules that aberrantly expressed in VS compared with normal tissues; 3) drug research for VS treatment. Studies were excluded, if they 1) merely found some imaging manifestations or clinical features related to VS growth; 2) case reports.

## 3 Results

We summarized the search results into five major types of biomarkers, which are summarized in **Table 1**.

**Table 1:** Summary table of potential biomarkers related to VS growth.

Major types	Action mechanisms	Biomarkers and their characteristics	References
Merlin pathway proteins	RTK signal proteins	VEGF expression was increased in VS, and anti-VEGF therapy was effective for NF2-VS.	(16)
		Pharmaceutical inhibition of PDGFR could reduce VS growth rate.	(17, 18)
		bFGF promoted proliferation and invasion of VS cells, and also might be a hearing protector in VS.	(16, 19)
		ErbB family proteins were aberrantly expressed in VS, and its inhibition has therapeutic effects on VS.	(20)
	Ras signal proteins	Merlin inhibits Ras signal transduction by interfering with GRB2 expression and inhibiting Rac1 and PAK1 activation.	(21–23)
	CPI-17–MYPT1 switch	CPI-17–MYPT1 switch was responsible for the conformational change of merlin. CPI-17 was over-expressed in VS.	(24–26)
	Hippo signal proteins	Merlin activated LATS1/2 and inhibited the destabilization of LATS1/2 by CRL4^DCAF1^. LATS1/2 promoted the degradation of YAP and inhibited the transcription of pro-proliferation and anti-apoptotic genes stimulated by YAP.	(27, 28)
Inflammatory signal	Local inflammation	The NF-κB signal activated in VS was considered to be the core of VS pathophysiology.	(29, 30)
		COX-2 was highly expressed in VS and was associated with high proliferation rate.	(31)
	Systemic inflammation	Macrophages, especially M2-type macrophages, were thought to promote VS growth.	(32, 33)
		High NLR was associated with VS growth.	(34)
Tumor miRNAs	Gene expression regulation	The upregulation of miR-29abc, miR-19, miR-340-5p, miR-21, miR-221 and downregulation of miR-744, let-7b were related to VS growth velocity.	(35, 36)
Tumor proteins	Glycoprotein	CD105 can be used as a MVD marker, and was correlated with VS size and growth rate.	(37)
	Neurotrophic factor	BDNF expression was correlated with mitotic activity in VS.	(38)
	Proteases	The expression of MMP-2 and MMP-9 was correlated with VS growth rate and was higher in cystic VS than solid VS. The proteolytic activity of MMP-14 was related to the degree of SNHL in VS patients.	(39–41)
		The expression of ADAM9 was higher in VS and related to functional impairment.	(42, 43)
CSF components	Mucopolysaccharide	HA levels were elevated in NF2-VS and correlated with the proliferation rate of schwannoma cells.	(44)
	Proteins	ABCA3, SCG1, KLF11, CA2D1, BASP1and PRDX2 were associated with VS growth.	(45)

### 3.1 Merlin Pathway Related Proteins

*Neurofibromin 2* gene mutation and the function loss of its transcription protein merlin (an acronym for moesin-ezrin-radixin-like protein) are widely regarded to play a paramount role in the pathogenesis of both sporadic and bilateral VS (46, 47). Merlin belongs to the ERM (for ezrin, radixin, moesin) family of cytoskeleton linker proteins and shares a common structural organization with it: a relatively conserved N-terminal FERM domain, followed by a α-helical region and a charged hydrophilic-COOH terminal tail (48). The *neurofibromin 2* gene encodes for two major isoforms of merlin: exon 16 skipping production isoform 1 and exon 16 retention isoform 2, both of which carry full tumor suppressive function (49).

Merlin maintains the stability of the cell membrane by the binding of integral membrane proteins and spectrin actin cytoskeleton and mediates cell contact inhibition (50). Merlin exerts its growth suppressive function by modulating the activity of intracellular promitogenic signal cascades related to tumor formation, including Ras/Raf/MEK/ERK (51), PI3K/Akt (52), c-JNK (53), Hippo signaling pathway, and the overall activity of the E3 ubiquitin ligase CRL4^DCAF1^ in the nucleus (54). Moreover, merlin was reported to assuming a possible role in the mediation of cell cycle progression (55).

**Figure 1:**
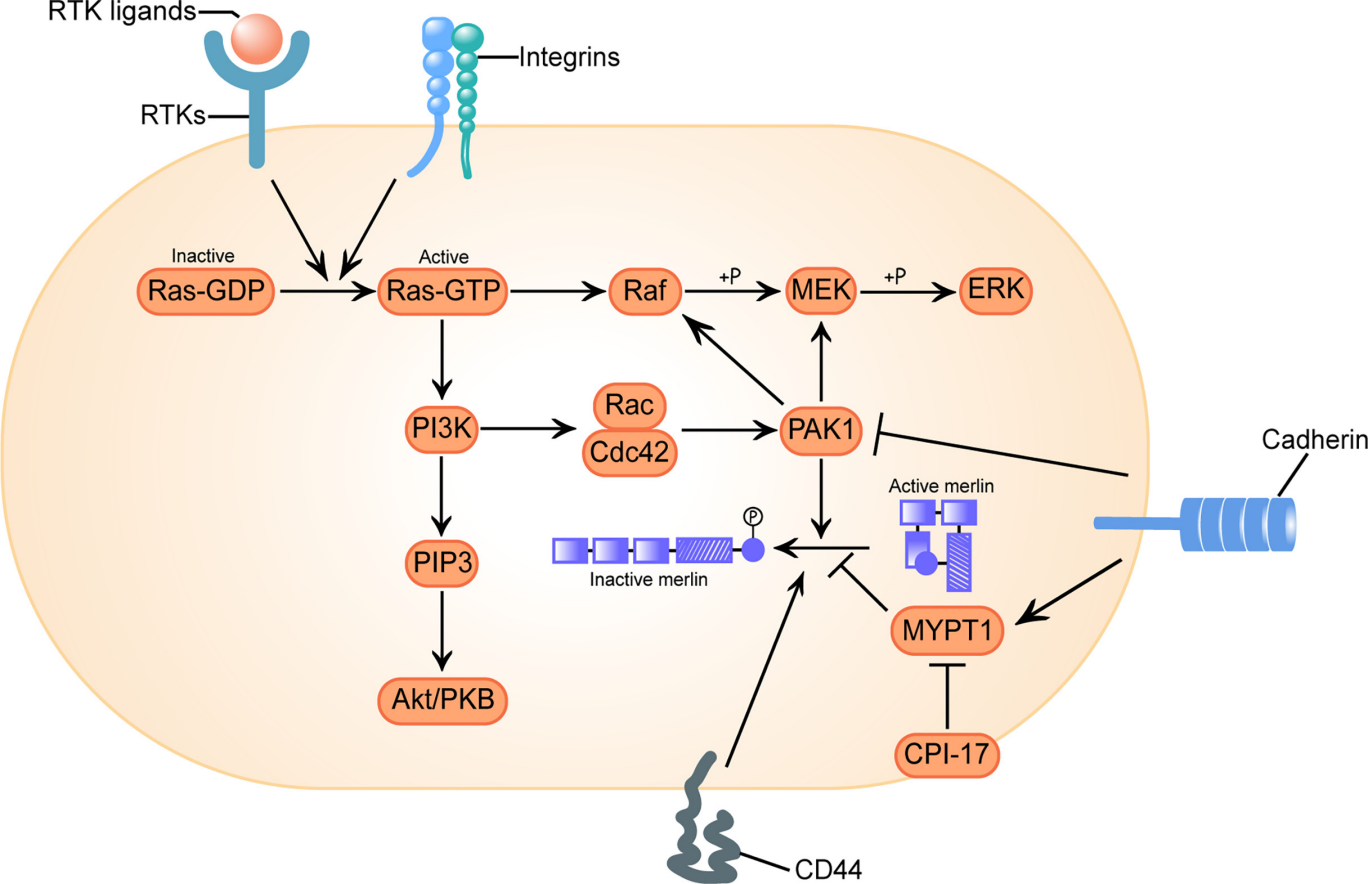
Configurational changes of Merlin in response to receptor tyrosine kinases (RTKs), integrins, clusters of differentiation 40 (CD40) and cadherins signal stimulation. Activation of RTKs and integrins triggers GTP loading of Ras, which drives three mitogenic downstream pathways: Raf/MEK/ERK, PI3K/Rac/PAK, and PI3K/PIP3/Akt/PKB. p21-activated kinase 1 (PAK1) is an effector of the downstream pathway activated by Ras, which phosphorylates merlin at Ser^518^ and converts it to an inactive conformation. Conversely, myosin phosphatase targeting subunit 1(MYPT1) dephosphorylates and reactivates merlin. C-kinase potentiated protein phosphatase-1 inhibitor of 17 kDa (CPI-17) acts as a cellular inhibitor of MYPT1, causes loss of function of merlin. CD44 inactivates merlin. Engagement of cadherins inactivates PAK1 and activates MYPT1, inducing merlin’s tumor suppressor function in a two-fold way. Here we adopt the broadly accepted hypothesis that phosphorylated merlin is the inactive form.

Classically, it was considered that the activity of merlin was regulated by phosphorylation on the main regulatory Ser^518^: dephosphorylated merlin functions as a growth inhibitor in a closed conformation formed by intramolecular association of its N-terminal domain (NTD) and carboxy-terminal domain (CTD), while phosphorylated merlin cannot form a folded state and is functionally deficient (56). However, subsequent researches have shown that transcripts of the mutated *neurofibromin 2* gene or phosphorylated merlin have a more closed form, leading to impaired contact-dependent inhibition of proliferation (57, 58). There is also a controversial view holding that merlin’s tumor suppressive function is independent of its conformational change (59). The activation of receptor tyrosine kinases (RTKs), integrins, CD44 and cadherin signal are responsible for the regulation of merlin’s phosphorylation *via* the downstream effector p21-activated kinase 1 (PAK1) and myosin phosphatase targeting subunit 1 (MYPT1) (**Figure 1**) (60, 61).

#### 3.1.1 RTKs

The over-expression and over-activation of at least four types of RTKs and their ligands involved in the progression or invasion of sporadic and NF2-associated VS: ErbB (62), platelet-derived growth factor (PDGF) (63), basic fibroblast growth factor (bFGF) (19) and vascular endothelial growth factor (VEGF) (64).

VEGF is one of the most prominent angiogenesis stimulator that can regulate irregular blood vessel sprouting and growth except for simple remodeling of the capillary basement membrane (65), and lead to development of an immuno suppressive tumor microenvironment (66). Compared with normal vestibular nerve, the expression of VEGF, VEGFR-1/Flt and VEGFR-2/Flk as well as the coreceptor NP1 were considerable increased in VS tissues, suggesting at least a part of neoplastic growth was induced *via* the promotion of angiogenesis (39, 64, 67). Tissue microarray analysis of 182 sporadic VSs found significantly higher VEGF levels in the groups of recurrent and preoperatively irradiated tumors when compared to primary VS patients (67). However, there are contradictory views as to whether VEGF expression is correlated with sporadic VS growth characteristics. Koutsimpelas et al. initially found a positive correlation between VEGF expression and tumor volume, tumor growth index (calculated by dividing the maximal tumor diameter by the patient’s age) and microvessel density (MVD, defined by CD31 staining) (16), but further investigation with expanded sample size demonstrated that although tumors with high proliferation index and high levels of VEGF and its receptor were more common than those with low proliferation activity expressing low levels of VEGF and its receptor, the expression of neither VEGF nor its receptors correlated with the proliferation indexes (Ki-67) or the growth characteristics of the tumors (67). Multiple anti-VEGF therapy studies in patients with progressive NF2-VS have shown that bevacizumab can not only ameliorate hearing loss and reduce tumor size (68), but also normalize the tumor vasculature and reduce vasogenic edema, which improved the delivery of oxygenation, a potent radiosensitizer, and therefore reduced radiation dose and minimized radiation-related neurotoxicity (69).

As a regulator of cell growth and division, PDGF can regulate stemness in schwannoma cell lines and have a role in tumorigenesis (70). The PDGF family consists of five different disulphide-linked dimers built up of four different polypeptide chains encoded by four different genes. These isoforms, PDGF-AA, PDGF-AB, PDGF-BB, PDGF-CC and PDGF-DD, act *via* two RTKs, PDGF receptors α and β (71). Nilotinib (17) and Gleevec (18, 63, 72) pharmacologically inhibit these two receptors and their main downstream signaling pathways that have been proved to be over-expressed and activated in VS, and could also potentially reduce the angiogenic activity and growth rate of VS.

bFGF (also known as FGF-2 or FGF-β) was not only an identified angiogenic cytokine (73), but also implicated in tumor maintenance and metastasis (74). bFGF was identified as a mediator to protect auditory neurons from acoustic trauma and aminoglycoside ototoxicity, it was 3.5-fold higher in good hearing VS *versus* poor hearing VS (75), and its plasma concentration increased while patient’s hearing improved after bevacizumab regimen (76). bFGF is also a known mitogen that promotes the proliferation of cell cultures derived from sporadic VS and increased the invasive phenotype mediated by Akt and ERK in HEI-193 cells (19, 77). In sporadic VS, increased levels of bFGF were positively correlated with tumor volume, tumor growth index and MVD (16). The mechanism by which bFGF stimulates mitosis might divergent from the way it modulates hearing. This notion, together with the results of cytokine array analysis where levels of ErbB, a well-established growth modulator, are not correlate with hearing status, may partly explain the irrelevance of tumor growth rate or tumor volume with VS patients’ hearing outcome (75).

The ErbB family consists of four members: ErbB-1/EGFR/Her1, ErbB-2/Neu/Her2/p185, ErbB-3/Her3, and ErbB-4/Her4. ErbB family is upregulated in many malignant tumors, and also aberrantly expressed in VS. It has been reported that 68% of EGFR (62% sporadic and 75% NF2-associated VS), 84% of ErbB2 (76% sporadic and 94% NF2-related VS) and 34% of ErbB3 were upregulated in VS (20). Of EGFR ligands, EGF was up-regulated in all NF2-related VS, but none of the sporadic VS; Neuregulin was up-regulated in 86% of sporadic VS and 19% of NF2-related VS (20). In HEI-193 cells, the addition of EGF increased cellular invasion by 10-fold, which could be reduced by the inhibition of PI3K/Akt (19). Both dual small molecule inhibitor of EGFR and ErbB2 Lapatinib and the EGFR inhibitor Erlotinib demonstrated their therapeutic effects in VS (78–80).

#### 3.1.2 Ras and Related Raf/MEK/ERK, PI3K/Akt and Rac/PAK

The small G-protein Ras, the downstream oncoprotein of RTKs that cycles between an inactive GDP-bound and an active GTP-bound state, play a role in the cascade of cell proliferation and division. In its active state, Ras can interact with several different effectors, thereby triggering an array of downstream signaling networks that responsible for promoting cellular transformation and driving tumorigenesis, such as Ras/Raf/MEK/ERK and Ras/PI3K/Akt pathways (81–83). It is well known that activation of Ras leads to subsequent activation of Rac/Cdc42 and its downstream effector PAK1, whose phosphorylation of Raf on Ser^338^ and MEK on Ser^298^ is required for effective signal transfer of Ras (**Figure 1**) (84, 85).

An increasing body of literature suggests that merlin counteract Ras-induced transformation at multiple levels. Merlin interferes with the expression of endogenous growth factor receptor binding 2 (GRB2) protein (21), directly reduces the GTP-loading of Ras and Rac to restrain their activation (86), and directly binds to the p21 binding domain (PBD) of PAK1 to interrupt PAK1 activation and its recruitment to focal adhesions (22, 87). Merlin competitively binds to angiomotin and releases the Rac1 negative regulator Rich1, which ultimately attenuates Rac1 signaling (23). Dysfunction of merlin would allow for enhanced Ras signal transduction and accelerated tumor growth rate (24).

Merlin itself is regulated by PAK reciprocally, it was phosphorylated at Ser^518^ by active PAK and therefore lose the inhibition of cell transformation. Activated Rac expression induces merlin phosphorylation and decreases the association between merlin and cytoskeleton (60). Based on the study of the role of Ras pathway signal transduction in the growth of VS, preclinical assessment of PAK inhibitors (88), MEK1/2 inhibitors (81) and Ailanthone, the down regulator of Ras and Raf, all exhibited certain antitumor properties (89).

#### 3.1.3 MYPT1

MYPT1 is a phosphatase that forms the C-kinase potentiated protein phosphatase-1 inhibitor of 17 kDa (CPI-17)–MYPT1 switch along with its most specific and potent inhibitor CPI-17, regulating the phosphorylation of both merlin and other ERM family proteins (24, 90). Although highly homologous, the activity changes of merlin and other ERM proteins after C-terminal phosphorylation are opposite, and fulfils an antagonistic role in Ras activity control: the phosphorylation inactivates merlin, which counteracts Ras-induced transformation (91), but activates ERM proteins, which are essential for proper Ras activation (92). Thus, the oncogenic protein CPI-17 may activate Ras signaling in a two-fold way.

A previous systematically investigation on schwannomas showed that CPI-17 stained negative in non-tumor pathologies, but specifically up-regulated in over 90% of schwannomas, primarily in sporadic schwannomas (25). Moreover, high CPI-17 levels were found to correlate with higher Ki-67 proliferation indices, indicating a putative role of CPI-17 in schwannoma progression (25). Xu et al. found the over-expression of CPI-17 was a prominent feature of sporadic VS tissues, and there was a significantly positive correlation between CPI-17 expression and merlin phosphorylation (26), confirming the rationality of the causal chain CPI-17–MYPT1–merlin dysfunction in VS.

#### 3.1.4 Yes-Associated Protein (YAP)

**Figure 2:**
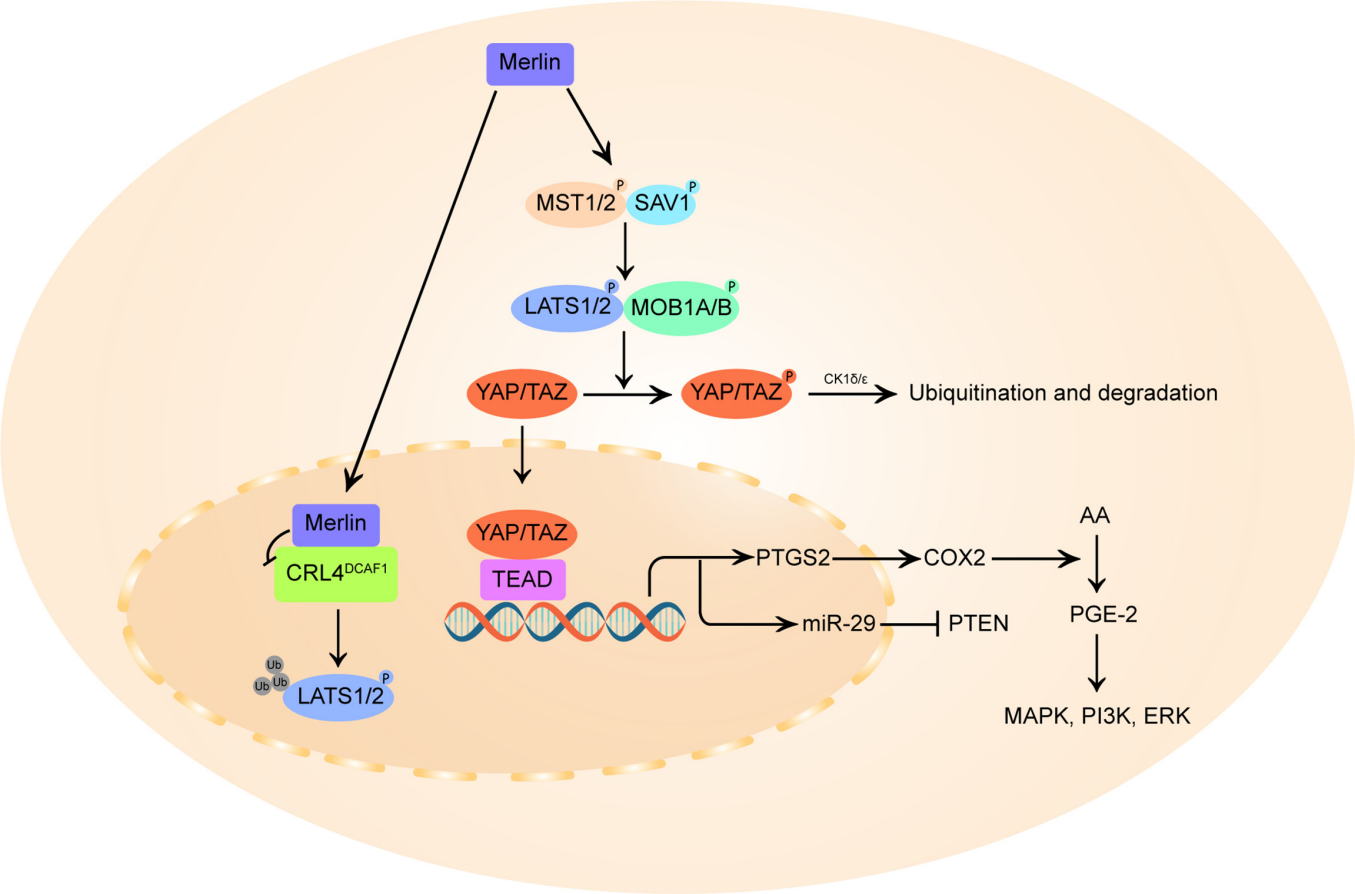
Schematic diagram of merlin and Hippo pathway signaling. On the one hand, merlin initiates the Hippo pathway by directly activating mammalian STE20-like protein (MST1/2), which in turn phosphorylates large tumor suppressor homolog 1/2 (LATS1/2), or by recruiting LATS 1/2 to the plasma membrane for phosphorylation by MST1/2 kinases. The activated LATS1/2 directly phosphorylates Yes-associated protein (YAP) at Ser^397^ and transcriptional coactivator with PDZ-binding motif (TAZ) at Ser^311^, priming the neighboring phosphodegron motif for phosphorylation by CK1δ/ϵ kinases and multi-level control of YAP/TAZ levels. In parallel, merlin also translocates to the nucleus and blocks the activity of the nuclear E3 ubiquitin ligase CRL4^DCAF1^, which promotes the ubiquitylation of LATS1/2 to activate YAP. In merlin-deficient cells, YAP/TAZ accumulates in the nucleus and forms hybrid transcription factors with TEA domain (TEAD) family proteins to promote the transcription of pro-proliferative genes, including PTGS2 and miR-29. PTGS2 encodes cyclooxygenase-2 (COX-2) that catalyzes the conversion of arachidonic acid (AA) to prostaglandin E-2 (PGE-2), thus promoting PGE-2 mediated MAPK, ERK, PI3K pathway. By inducing miR-29, YAP can also inhibit the translation of phosphatase and tensin homolog (PTEN), a broadly downregulated tumor suppressor in vestibular schwannoma.

The deregulation of the Hippo pathway and the accompanying activation of Yes-associated protein (YAP) has been implicated in VS cell proliferation (27). As described in **Figure 2**, merlin initiate Hippo pathway to promote the phosphorylation and degradation of YAP and its homologous protein transcriptional coactivator with PDZ-binding motif (TAZ) (93, 94). In the inactivated state of merlin, YAP/TAZ migrates into the nucleus and binds to TEA domain family members (TEAD), stimulating the transcription of pro-proliferation and anti-apoptotic genes (27, 95).

The E3 ubiquitin ligase CRL4^DCAF1^ can directly ubiquitinylate and destabilize LATS1/2 to activate YAP (54). Merlin can translocates into the nucleus, binds with high affinity to CRL4^DCAF1^ and blocks its function (28). It is postulated that this merlin-CRL4^DCAF1^-LATS1/2-YAP signaling axis might be the key mechanism of merlin’s tumor suppressive effect. Although no studies have directly confirmed the correlation between VS tumorigenesis and CRL4^DCAF1^ activity, targeted inhibition of the upstream activator of CRL4^DCAF1^, NEDD8-activating enzyme (NAE), can induce inhibitory YAP phosphorylation and reduce cell proliferation in mouse *neurofibromin 2*-mutant schwannoma cells and human *neurofibromin 2*-mutant mesothelioma cell line (96), indicating a growth-promoting role of CRL4^DCAF1^ in *neurofibromin 2*-inactivated tumors.

### 3.2 Inflammatory Signal

At present, there is increasing evidence that a variety of solid tumors contain a certain degree of tumor-associated inflammation that play a role in the cancer formation, progression and metastasis (97). Inflammatory response in cancer patients comprises both the local inflammatory response and the systemic response. The local inflammatory response is mediated by chemokines, cytokines, growth factors and matrix metalloproteinases (MMPs) secreted from *in situ* tumor cells, stromal cells, and infiltrating immune cells, and can became accomplices to cancer development by enhancing cell survival, invasion, neovascularization, and adaptive immunity suppression (98). Meanwhile, inflammation in the tumor microenvironment could be reflected in peripheral circulation, i.e., systemic inflammatory response that characterized by white cell components in peripheral blood, such as the platelet−to−lymphocyte ratio (PLR) and neutrophil−to−lymphocyte ratio (NLR), which have been highlighted as probable markers of pathologic responses for numerous types of solid tumors (99, 100).

It has been demonstrated that the volume increase of VS is not merely based on cell proliferation, but is also implicated with (neo)vascularization, intratumoral hemorrhage, cyst formation and inflammatory reaction (32, 101, 102).

#### 3.2.1 Nuclear Factor-Kappa B (NF-κB)

**Figure 3:**
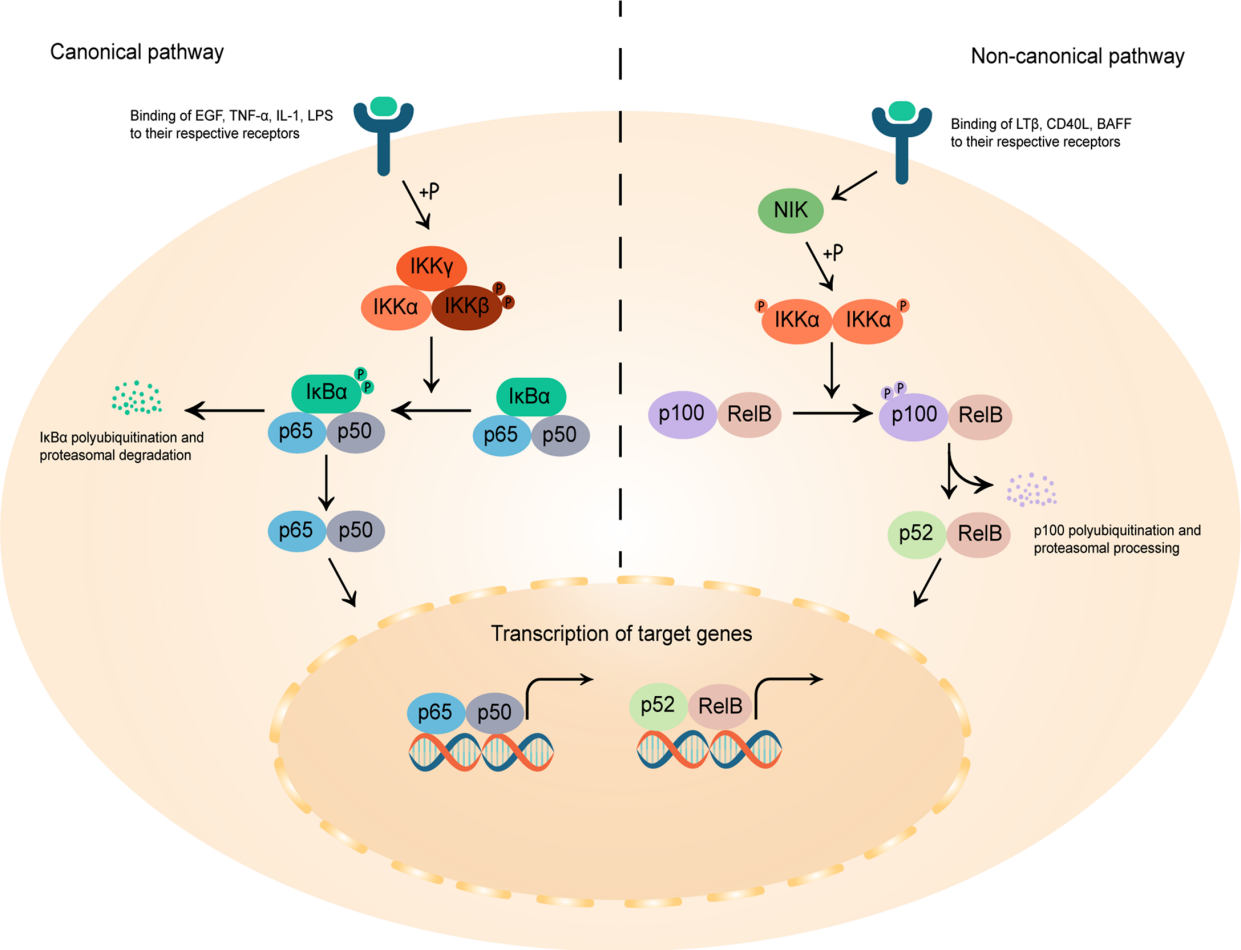
Schematic diagram of the canonical and non-canonical NF-κB signaling pathway. In the canonical pathway, on the left, the binding of epidermal growth factor (EGF), tumor necrosis factor-α (TNF-α), interleukin-1 (IL-1) and lipopolysaccharides (LPS) to their respective receptors lead to the phosphorylation of inhibitor of κB (IκB)-kinase β (IKKβ) in the IKK complex, which in turn phosphorylates IκBα, culminating in its polyubiquitination and proteasomal degradation. The free NF-κB homo- or heterodimers, in this case p65/p50, then translocate into the nucleus to bind to κB motif and promote target gene transcription. The non-canonical pathway, on the right, is activated by the binding of lymphtoxin β (LTβ), CD40 ligand (CD40L), and B-cell activating factor (BAFF) to their respective receptors, causing IKKα dimers phosphorylation by the NF-κB-inducing kinase (NIK). Phosphorylation of p100 by activated IKKα initiates proteasomal processing of p100 N-terminal to form p52. The p52/RelB heterodimers can then undergoes nuclear translocation and modulates the gene transcription of pro-inflammatory cytokines in a feedback-loop way.

NF-κB is a ubiquitous, evolutionary conserved transcription factor central to cell growth, apoptosis, inflammation and various malignant diseases (103). As a group of homo- and hetero-dimeric proteins composed of members of the Rel family, NF-κB complexes includes p65/RelA, RelB, c-Rel, p50/p105 (NF-κB1), and p52/p100 (NF-κB2) five subunits (104), and resides in the cytoplasm of non-stimulating cells in the form of complexes with inhibitor of κB (IκB). The NF-κB signaling pathway is activated by inflammatory cytokines or growth factors, and induces gene transcription of these factors in a feedback-loop way (**Figure 3**) (105–107).

Studies have shown that merlin negatively regulates NF-κB signaling, and the elevated NF-κB signal was considered as the hub of the interaction network of aberrant expressed molecules in VS pathobiology (29, 30, 108). In the absence of merlin, overexpressed NIK and IKKα induced high NF-κB dependent transcription (29), while merlin expression blocks NF-κB activation by the inhibition of p65, NIK, IKKα, tumor necrosis factor-α (TNF-α)-induced IκB degradation, NF-κB–DNA binding and endogenous NF-κB signaling (30). Besides, activated NF-κB is thought to be the mechanism by which elevated p75^NTR^ expression promotes cell survival in VS (109). Hyper NF-κB activity in VS potentiated hepatocyte growth factor (HGF) to c-Met autocrine feed-forward loop to promote tumor cell proliferation (108), and increased the expression of factors that reported to be increased in VS or correlated with the prognosis or absolute tumor growth rate of VS, such as matrix metalloproteinase 2 (MMP-2), MMP-9 (40), MMP-14 (41), COX-2 (110), interleukin-1 (IL-1), IL-6, TNF-α (64) and signal transducers and activators of transcription 1 (STAT1) (111).

#### 3.2.2 Cyclooxygenase-2 (COX-2)

COX-2 is an isozyme of the COX family, catalyzes the conversion of arachidonic acid to prostaglandins, including the biosynthesis of prostaglandin E-2 (PGE-2). In a healthy state, COX-2 is involved in maintaining cell homeostasis, while its response to homeostatic dysregulation might lead to the development of cancer (112). Studies have demonstrated that COX-2 is upregulated in various solid tumors and involved in cancer inflammation (113). COX-2 is released to the cancer microenvironment by type II macrophages, cancer−associated fibroblasts and tumor cells, and can suppress tumor cell apoptosis, enhance cell adhesion to promote tumor-induced angiogenesis and achieve an aggressive phenotype (114, 115).

COX-2 presented at high levels in majority (96.67%) of VS tissue samples, and those VSs with higher COX-2 expression showed higher proliferation rate (31). Transcription of COX-2 is thought to be promoted by NF-κB. Aspirin modifies both NF-κB signaling and COX-2 expression (116), and has been shown to have therapeutic effects in several tumors with high COX-2 levels (117, 118). Although some studies have shown that aspirin may halt VS growth (119, 120), the latest meta-analysis disproved this viewpoint (121).

#### 3.2.3 Macrophages

Tumor associated macrophages (TAMs) can be broadly classified into two categories, both of which are differentiated from monocytes in response to stimulus signals (122). The first type is M1-type inflammatory macrophages, also known as classically activated macrophages. M1-type macrophages are critically important in host defense and killing of tumor cells by the production of pro-inflammatory cytokines such as interferon-γ (IFN-γ), TNF-α and IL-18, which have potent microbiome-killing properties and hence are considered as ‘good’ macrophages (123). In order to avoid collateral damage to healthy cells/tissues by M1-type macrophages, M2-type macrophages, or alternatively activated macrophages, usually presented at the later stage of inflammatory response to suppressing destructive immunity, promoting wound repair and fibrosis. The effects of M2-type macrophages are manifested in the tumor microenvironment as inhibiting anti-tumor immunity, promoting tumor matrix remodeling and inducing angiogenesis (123). Collective data have shown that the high densities of M2-type macrophages in the tumor microenvironment are associated with the secretion of VEGF and MMP-9 (124), and worse prognosis in numerous cancer types (125, 126).

In a previous study, it was demonstrated that macrophages, rather than tumor cells, accounted for the majority of proliferating cells in the growing sporadic VS (32). Using Iba-1 as a pan-macrophage marker, the study of 24 sporadic VSs and 20 NF2-related VSs showed that the microvessel surface area was positively correlated with TAM density, and that Iba-1+ macrophages contributed significantly to VEGF production (127). This supports the idea that targeting macrophages along with vascular supply should be viewed as promising therapeutic options in both VS groups.

de Vries et al. determined the role of M2-type macrophages in promoting angiogenesis and volumetric tumor growth in sporadic VS tissue sections. They compared the expression of CD163, a specific marker for M2-type macrophages, in 10 rapidly growing VSs and 10 slow-growing VSs, and found that CD163 expression was significantly higher in fast-growing VS, and tumors with higher CD163 expression had more microvessels (as assessed by CD31). Consistent with these findings, the levels of macrophage colony-stimulating factor (M-CSF), an important cytokine that stimulates macrophage polarization into an M2-type macrophage, and its synergistic cytokine interleukin-34 (IL-34), are increased in fast-growing VS, suggesting their potential function as promoters of tumor progression (33). Recently, Bi et al. demonstrated variable expression of immune regulatory markers as well as immune infiltrates in VS (128). They found both tumor volume and volumetric growth were positively correlated with CD163 expression, and the relationship between PD-L1 and growth strengthened with increasing CD163 infiltration, suggesting a prominent role of immunotherapy in VS treatment (128).

#### 3.2.4 Neutrophil-to-Lymphocyte Ratio (NLR)

As a representative index of systemic inflammation, preoperative peripheral blood NLR is related to the pathological characteristics of many tumors (129).The pretreatment NLR is calculated according to the absolute neutrophil count (ANC) and the absolute lymphocyte count (ALC) of routine blood tests obtained prior to any intervention. A high NLR indicated greater systemic inflammation and elevated circulating concentrations of pro-inflammatory and angiogenic cytokines, which can enhance tumor progression, immunosuppression and peritumoral stroma formation, and therefore is considered as a promising negative prognostic biomarker in solid tumors (130).

The utility of NLR has been pointed for predicting oncological growth in patients with VS. Kontorinis et al. found a significant difference of NLR between 79 growing VSs and 82 non-growing VSs, with high NLR was observed predominantly in the growing VS group (34). However, there is no such discrepancy between regressing VS and growing VS (131). A probable speculation is that NLR-associated inflammation functions not only in the pathomechanism of tumor growth, but also in the decrease of tumor size. Currently, the critical limitation needs to be obviated for NLR’s clinical application and further investigation is that there is no consensus on the cutoff value of NLR (34).

### 3.3 miRNAs

miRNAs are small non-coding RNAs that responsible for the post-transcriptional regulation of genes by partially or perfectly complementary to the target mRNAs, leading to protein synthesis abortion or transcription inhibition (132). miRNAs play an important role in tumor initiation and development, and are potential targets for therapeutic interventions in diverse cancers.

Several differentially expressed miRNAs have been identified in VS tissues (133, 134). Using principal component analysis, affected gene ontology analysis, and analysis of miRNA expression fold changes, Sass et al. determined the relationship between rapid tumor growth and upregulation of miR-29abc, miR-19, miR-340-5p, miR-21, miR-221 and downregulation of miR-744 and let-7b (35). miR-29 fulfills controversial function in tumor progression and invasion in different tumors (135–137), and was reported to be upregulated in previous VS studies (134). Concordant with this research, Cioffi et al. demonstrated that miR-21 was over-expressed in VS compared with normal vestibular nerve tissue (36). Yang et al. found that downregulation of miR-21 by ailanthone reduced the proliferative potential of VS cells and induced apoptosis and autophagy (89). Other deregulated miRNAs also serve as modulators of tumor-related signaling pathways, such as miR-340 in Ras/Raf/MAPK (138) and let-7b in SOCS1/STAT (139). Notably, the contributions of miR-19, miR-21 and miR-221 to tumor growth are all related to their down-regulation of tumor suppressor PTEN (36, 136, 140), suggesting the role of PTEN and its downstream PI3K/Akt/mTOR signaling pathway in VS cancerigenic process.

### 3.4 Proteins in Tumor Tissue

CD105, better known as endoglin, is a homodimeric transmembrane glycoprotein that highly expressed on activated angiogenic endothelial cells (141). Used as a marker of MVD, CD105 has proven itself as a potent prognostic indicator and a potential target for antiangiogenic therapy and chimeric antigen receptor-based T-cell (CAR-T) immunotherapy in several tumors (142–144). Calculated by immunohistochemically assessed CD105 expression, Gino et al. correlated the vessel cross-sectional area (VA) and vessel density (VD) with tumor dimensions and tumor growth rate in both NF2-associated VS and sporadic VS, and found a positive correlation between VD and tumor growth rate in NF2-VS, and VA and VD with tumor size in sporadic VS (37). Considering the great achievements of anti-angiogenesis therapy in VS and the specificity of CD105 to indicate the vascular front where sprouting takes place, it seems reasonable to use circulating CD105 to monitor tumor growth and to treat VS with anti-endoglin antibodies.

With several over-expressed neurotrophic factors in VS drawn attention as putative key mediators of tumor growth, Kramer et al. explored the relationship between gene expression profiles of neurotrophic factors and proliferation-associated Ki-67 labelling index, and observed significantly elevated brain derived neurotrophic factor (BDNF) expression that correlated with mitotic activity in VS (38).BDNF plays a pivotal role in myelination processing and supports the survival and synaptic integrity of the auditory nerve (145). This mitosis-promoting neurotrophic factor, together with the previously mentioned bFGF, which has the dual effects of hearing protection and growth stimulation of VS cells (16, 75), may partially explain the seemingly paradoxical phenomenon that VS patients with tumor growth do not necessarily experience hearing loss.

MMPs comprise a large family of zinc- and calcium-dependent proteolytic enzymes. Apart from their essential role in promoting cell differentiation and the reconstruction of the extracellular matrix in a regenerative milieu, MMPs play a fundamental role in tumor progression, invasion, and angiogenesis (146). Expression characteristics of several MMPs family members in VS have been explored. MMP-2 and its endogenous inhibitors tissue inhibitors of metalloproteinase (TIMP)-1 were found interstitially in sporadic solid VS, while the majority of MMP-9 was localized in tumor cells, and its concentration in tumor sample homogenates was positively correlated with the absolute tumor growth rate (40). Consistent with previous studies showing that the proteolytic activity of MMPs might degrade collagen, fibronectin, laminin and contribute to cyst formation and expansion, the expression of MMP-2 and MMP-9 were upregulated in cystic VS samples compared with solid VS (147). In cystic VS tissues, MMP-2 was localized to tumor cells on the cyst cavity inner surface and its levels were higher in the cystic fluid than in other samples (148).

Histologically, VS can be classified into two tissue types, where Antoni type A has a compact structure with interwoven bundles of long bipolar spindle cells and Antoni type B characterized by a loose texture and small, uniform satellite cells. It is known that cystic VS consist of a large mass of Antoni type B area, where the expression of MMP-14 was significantly higher than that in Antoni type A (39). The abundance and proteolytic activity of MMP-14 in VS patients was correlated with the degree of SNHL and surgical outcomes (41). Besides, MMP-2, MMP-9 and MMP-14 are all principal proteins of the MMPs family in the vasculature, implying degeneration of tumor tissues by MMPs and the accompanying increased tumor vessel permeability and adhesion to the facial nerve are critical in VS tumorigenesis, cyst formation and preoperative hearing impairment.

As a membrane-anchored protein of the A-Disintegrin and Metalloproteinase (ADAM) protein family, the overexpression of ADAM9 in solid tumors has been correlated with aggressive tumor phenotypes and unfavorable clinical prognosis (149). ADAM9 may implicated in tumor progression and invasion either *via* non-proteolytic mechanisms that include interactions between tumor cells and endothelial or peritumoral stromal cells (150), or proteolytic mechanisms that involve an enzymatic modification called “shedding” or processing of cell-surface proteins (151). The value of ADAM9 inhibition in reducing migration and invasion of different solid tumors has been established (152, 153). The earliest expression assessment of ADAM9 in VS by Breun et al. found an 8.8-fold higher ADAM9 mRNA level in VS compared with in healthy peripheral nerves, and a strong correlation between ADAM9 mRNA expression and the degree of functional impairment (42). Further, in VS primary cell cultures, ADAM9 knock down caused a 58% reduction in cell numbers, which might be a hint that ADAM9 plays a role in inducing VS progression (43).

### 3.5 Components in Cerebrospinal Fluid

VSs usually originate from Schwann cells of the cisternal portion of nerve rootlets exposed to CSF and may alter CSF composition by secreting proteins or altering CSF metabolism (154). CSF-based liquid biopsy approaches have been used to characterize protein expression during the pathogenesis of several brain tumors (155, 156), and differential components analyses of CSF are also expected to yielded diagnostic and therapeutic targets in VS.

Two studies have been done on CSF composition to identify biomarkers that predict VS growth. Ariyannur et al. found the levels of hyaluronan (HA) were 17-fold higher in NF2-related VS cases compared to the controls, and the rate of HA synthesis and secretion by primary schwannoma cells was commensurate with their proliferation rate (44). HA is a cell-surrounding mucopolysaccharide that binds to the Schwann cell surface CD44 receptor to trigger an uninterrupted cell proliferation cascade. Deranged HA-CD44 interaction has been identified as one of the central causative factors for schwannoma and a tumor suppressor target of merlin (157). Schwannoma cells increase their self-reproductive potential by secreting HA under the innate condition, suggesting that elevated HA in the CSF of patients with NF2-VS may be used as an indicator of tumor growth.

By characterizing the CSF proteome among VS patients with different grades, Huang et al. found that ATP-binding cassette subfamily A member 3 (ABCA3), secretogranin-1 (SCG1), Krueppel-like factor 11 (KLF11), voltage-dependent calcium channel subunit alpha-2/delta-1 (CA2D1), brain acid soluble protein 1 (BASP1), and peroxiredoxin-2 (PRDX2) in CSF were associated with VS growth (45). It is notable that some of them did not simply increase or decrease as the tumor grows, but reached their maximum or minimum values at certain phases, suggesting that volumetric growth of VS may be driven by different signaling pathways at different tumor stages.

## 4 Discussion

VS is a benign but potentially devastating tumor whose aggressively growth can be associated with significant morbidity including deafness and facial neuropathy. Usually, treatment of VS relies on surgery, radiation and regular follow-up. Better knowledge of the pathogenesis of VS has led to several targeted therapies with the effect of reducing tumor volume or restoring patients’ hearing, of which bevacizumab has achieved the most promising results (68).

The majority of VSs may not enlarge after initial diagnosis, with an average annual growth rate of 1.11 mm (9). In order to preserve hearing function and enhance the quality of life, a substantial proportion of newly diagnosed patients will choose wait-and-scan policy, who were put at the risk of sudden tumor growth due to extremely variable growth patterns of VS. Hence, it would be helpful to identify predictive factors for VS growth at time of diagnosis. Several epidemiological, clinical, and radiological characteristics were thought to be related to the increased risk of subsequent tumor growth, such as patients’ age, tumor location, hearing loss, and grow at first follow-up (9, 12–14). But there were some inconsistencies among reports of predicting growth-related factors (9), making the accuracy of this approach debatable.

From the perspective of VS growth mechanisms, numerous achievements had been obtained in exploring the relationship between tumor growth and biomarkers. The exact mechanism of VS onset and progression has remained elusive, with only a broad association with genetic and epigenetic aberrations of merlin, inflammation factors, proteolytic enzymes and nutrient supply pathways. Notably, there are mutual interactions between abnormally expressed molecules in VS biology. Merlin, whose conformational changes are regulated by RTKs and integrin signals, is also an inhibitor of the pro-inflammatory transcription factor NF-κB (30). On the other hand, high numbers of tumor infiltrating leukocytes in VS tissues can enhance cell survival, promote tumor growth and degenerative changes through the production of growth factors, cytokines, and MMPs (33, 128, 158). These evidences indicate that the progression of VS appears to be the result of interactions of these dysregulated pathways, drugs that target multiple signaling pathways simultaneously merit further investigation.

Despite the many recent advances in the identification of growth-related biomarkers, their translational researches have encountered several problems. First, current available VS animal models have some limitations. The commonly utilized animal models for the study of VS including patient-derived xenograft mice model (159), merlin-deficient Schwann cell or SC4 cells mouse model (160, 161). Nevertheless, merlin-deficient Schwann cells may not accurately reproduce VS (162), and it takes more than 2 weeks for the construction of mouse model. Recently proposed zebrafish xenograft model with shorter period of model establishment (163), as well as the application of 3D *in vitro* cell culture system (164) and *ex vivo* organ cultures (165) that fully mirror the growth patterns of VS, promise to further elucidate the role of these biomolecules in the pathogenesis of VS. Second, clinical validation of these hypotheses should take tumor diameter and clinical course into consideration. Current studies demonstrate non-linear changes of protein expression as VS size increases (45), suggesting that tumor progression at different grades might be stimulated by different signaling pathways and can therefore be predicted by different biomarkers. Along similar lines, different stages of the natural course of VS might be driven by different mechanisms either. During the enlarge of VS, accelerated growth, regression, or quiescence of the tumor can occur at any stage (2). Classifying patients according to the growth characteristics of VS and analyzing biomarker levels in different groups would shed light on which pathway induces VS initiation and which involves in VS shrinkage or growth cessation. Third, research is urgently needed to address how to obtain tissue expression data in a non-traumatic way. MRI texture analysis have been previously associated with VEGF expression in head and neck squamous cell carcinoma (166), and might also reflected VEGF levels in VS. A more precise non-invasive *in vivo* marker expression evaluation technique is targeted molecular imaging [MRI, computed tomography (CT), positron emission computed tomography/single-photon emission computed tomography (PET/SPECT) and fluorescence] (161, 167–169). Recently, Morrison et al. injected the covalently linked compounds formed by anti-VEGFR2 or anti-Her2/Neu monoclonal antibodies and near-infrared probe into Schwann cell xenograft models in mice, and observed strong correlations between day 1 tumor fluorescence and eventual maximum tumor volume (170). Apart from advanced imaging techniques, the circulating biomarkers level could also serve as convenient and reliable indicators for the prediction of subsequent tumor growth (37, 44).

We highlight some of the known and novel indicators with potential to predict VS growth enlightened therapeutic targets of VS and are critical for clinicians in stratifying patient’s risk preoperatively, formulating individualized therapeutic tactics, and guiding clinical trial enrollment.

## Author Contributions

YZ and JL wrote the first draft of the paper. JR and XH revised the draft. PZ and BW provided approval for publication of this review. All authors contributed to the article and approved the submitted version.

## Funding

This study was sponsored by Shanghai Science and Technology Committee Youth Sailing Program (19YF1405700), Clinical Research Plan of Shenkang Hospital Development Center (SHDC2020CR1049B), Chinese Academy of Medical Sciences Innovation Fund for Medical Sciences (2019-I2M-5-008), National Natural Science Foundation of China (NSFC81872938, 82003864).

## Conflict of Interest

The authors declare that the research was conducted in the absence of any commercial or financial relationships that could be construed as a potential conflict of interest.

## Publisher’s Note

All claims expressed in this article are solely those of the authors and do not necessarily represent those of their affiliated organizations, or those of the publisher, the editors and the reviewers. Any product that may be evaluated in this article, or claim that may be made by its manufacturer, is not guaranteed or endorsed by the publisher.

## References

[1] Jia H Lahlou G Wu H Sterkers O Kalamarides M. Management of Neurofibromatosis Type 2 Associated Vestibular Schwannomas. Curr Otorhinolaryngol Rep (2021) 9(2):170–6. doi: 10.1007/s40136-021-00341-x

[2] Tan D Killeen DE Kutz JW. The Natural History of Vestibular Schwannoma and When to Intervene. Curr Otorhinolaryngol Rep (2021) 9(2):134–8. doi: 10.1007/s40136-021-00337-7

[3] Huang X Xu J Xu M Zhou L-F Zhang R Lang L. Clinical Features of Intracranial Vestibular Schwannomas. Oncol Lett (2013) 5(1):57–62. doi: 10.3892/ol.2012.1011 23255894 PMC3525418

[4] Halliday J Rutherford SA McCabe MG Evans DG. An Update on the Diagnosis and Treatment of Vestibular Schwannoma. Expert Rev Neurother (2018) 18(1):29–39. doi: 10.1080/14737175.2018.1399795 29088993

[5] Pandrangi VC Han AY Alonso JE Peng KA St John MA. An Update on Epidemiology and Management Trends of Vestibular Schwannomas. Otol Neurotol (2020) 41(3):411–7. doi: 10.1097/mao.0000000000002542 31939906

[6] Pritchard C Clapham L Davis A Lang DA Neil-Dwyer G. Psycho-Socio-Economic Outcomes in Acoustic Neuroma Patients and Their Carers Related to Tumour Size. Clin Otolaryngol (2004) 29(4):324–30. doi: 10.1111/j.1365-2273.2004.00822.x 15270817

[7] Hasegawa T Kida Y Kato T Iizuka H Kuramitsu S Yamamoto T. Long-Term Safety and Efficacy of Stereotactic Radiosurgery for Vestibular Schwannomas: Evaluation of 440 Patients More Than 10 Years After Treatment With Gamma Knife Surgery Clinical Article. J Neurosurg (2013) 118(3):557–65. doi: 10.3171/2012.10.Jns12523 23140152

[8] Suryanarayanan R Ramsden RT Saeed SR Aggarwal R King AT Rutherford SA. Vestibular Schwannoma: Role of Conservative Management. J Laryngol Otol (2010) 124(3):251–7. doi: 10.1017/s0022215109992362 20003606

[9] Paldor I Chen AS Kaye AH. Growth Rate of Vestibular Schwannoma. J Clin Neurosci (2016) 32:1–8. doi: 10.1016/j.jocn.2016.05.003 27450283

[10] Martin TPC Tzifa K Kowalski C Holder RL Walsh R Irving RM. Conservative *Versus* Primary Surgical Treatment of Acoustic Neuromas: A Comparison of Rates of Facial Nerve and Hearing Preservation. Clin Otolaryngol (2008) 33(3):228–35. doi: 10.1111/j.1749-4486.2008.01715.x 18559028

[11] Goshtasbi K Abouzari M Moshtaghi O Sahyouni R Sajjadi A Lin HW. The Changing Landscape of Vestibular Schwannoma Diagnosis and Management: A Cross-Sectional Study. Laryngoscope (2020) 130(2):482–6. doi: 10.1002/lary.27950 PMC7546694 30953401

[12] Kim JS Cho Y-S. Growth of Vestibular Schwannoma: Long-Term Follow-Up Study Using Survival Analysis. Acta Neurochir (2021)163(8):2237–45. doi: 10.1007/s00701-021-04870-8. in press. 34003365

[13] Roehm PC Gantz BJ. Management of Acoustic Neuromas in Patients 65 Years or Older. Otol Neurotol (2007) 28(5):708–14. doi: 10.1097/01.mao.0000281805.44197.ec 17667776

[14] Lee JD Park MK Kim JS Cho Y-S. The Factors Associated With Tumor Stability Observed With Conservative Management of Intracanalicular Vestibular Schwannoma. Otol Neurotol (2014) 35(5):918–21. doi: 10.1097/mao.0000000000000338 24686291

[15] Higuchi Y Ikegami S Horiguchi K Aoyagi K Nagano O Serizawa T. Predicting Potential of Rapid Tumor Growth in Small to Medium Vestibular Schwannomas on the Basis of Sway Assessed Using Posturography. World Neurosurg (2021) 148:E406–14. doi: 10.1016/j.wneu.2020.12.175 33444828

[16] Koutsimpelas D Stripf T Heinrich UR Mann WJ Brieger J. Expression of Vascular Endothelial Growth Factor and Basic Fibroblast Growth Factor in Sporadic Vestibular Schwannornas Correlates to Growth Characteristics. Otol Neurotol (2007) 28(8):1094–9. doi: 10.1097/MAO.0b013e31814b2787 17721409

[17] Sabha N Au K Agnihotri S Singh S Mangat R Guha A. Investigation of the In Vitro Therapeutic Efficacy of Nilotinib in Immortalized Human NF2-Null Vestibular Schwannoma Cells. PloS One (2012) 7(6):10. doi: 10.1371/journal.pone.0039412 PMC3379978 22745749

[18] Mukherjee J Kamnasaran D Balasubramaniam A Radovanovic I Zadeh G Kiehl TR. Human Schwannomas Express Activated Platelet-Derived Growth Factor Receptors and C-Kit and Are Growth Inhibited by Gleevec (Imatinib Mesylate). Cancer Res (2009) 69(12):5099–107. doi: 10.1158/0008-5472.can-08-4475 19509233

[19] Blair KJ Kiang A Wang-Rodriguez J Yu MA Doherty JK Ongkeko WM. EGF and bFGF Promote Invasion That Is Modulated by PI3/Akt Kinase and Erk in Vestibular Schwannoma. Otol Neurotol (2011) 32(2):308–14. doi: 10.1097/MAO.0b013e318206fc3d 21178801

[20] Doherty JK Ongkeko W Crawley B Andalibi A Ryan AF. ErbB and Nrg: Potential Molecular Targets for Vestibular Schwannoma Pharmacotherapy. Otol Neurotol (2008) 29(1):50–7. doi: 10.1097/mao.0b013e31815d4429 18199957

[21] Lim JY Kim H Jeun SS Kang SG Lee KJ. Merlin Inhibits Growth Hormone-Regulated Raf-ERKs Pathways by Binding to Grb2 Protein. Biochem Biophys Res Commun (2006) 340(4):1151–7. doi: 10.1016/j.bbrc.2005.12.122 16405865

[22] Kissil JL Wilker EW Johnson KC Eckman MS Yaffe MB Jacks T. Merlin, the Product of the Nf2 Tumor Suppressor Gene, the P21-Activated Is an Inhibitor of Kinase, Pak1. Mol Cell (2003) 12(4):841–9. doi: 10.1016/s1097-2765(03)00382-4 14580336

[23] Yi C Troutman S Fera D Stemmer-Rachamimov A Avila JL Christian N. A Tight Junction-Associated Merlin-Angiomotin Complex Mediates Merlin's Regulation of Mitogenic Signaling and Tumor Suppressive Functions. Cancer Cell (2011) 19(4):527–40. doi: 10.1016/j.ccr.2011.02.017 PMC3075552 21481793

[24] Jin HC Sperka T Herrlich P Morrison H. Tumorigenic Transformation by CPI-17 Through Inhibition of a Merlin Phosphatase. Nature (2006) 442(7102):576–9. doi: 10.1038/nature04856 16885985

[25] Hagel C Dornblut C Schulz A Wiehl U Friedrich RE Huckhagel T. The Putative Oncogene CPI-17 Is Up-Regulated in Schwannoma. Neuropathol Appl Neurobiol (2016) 42(7):664–8. doi: 10.1111/nan.12330 27248983

[26] Xu JH Zhang Y Shi YX Yin DM Dai PD Zhao WD. CPI-17 Overexpression and Its Correlation With the NF2 Mutation Spectrum in Sporadic Vestibular Schwannomas. Otol Neurotol (2020) 41(1):E94–102. doi: 10.1097/ma0.0000000000002430 31789805

[27] Zhao F Yang ZJ Chen Y Zhou QY Zhang J Liu J. Deregulation of the Hippo Pathway Promotes Tumor Cell Proliferation Through YAP Activity in Human Sporadic Vestibular Schwannoma. World Neurosurg (2018) 117:E269–79. doi: 10.1016/j.wneu.2018.06.010 29902598

[28] Li W Giancotti FG. Merlin's Tumor Suppression Linked to Inhibition of the E3 Ubiquitin Ligase CRL4 (Dcaf1). Cell Cycle (Georgetown Tex) (2010) 9(22):4433–6. doi: 10.4161/cc.9.22.13838 PMC3048042 21084862

[29] Dilwali S Briet MC Kao SY Fujita T Landegger LD Platt MP. Preclinical Validation of Anti-Nuclear Factor-Kappa B Therapy to Inhibit Human Vestibular Schwannoma Growth. Mol Oncol (2015) 9(7):1359–70. doi: 10.1016/j.molonc.2015.03.009 PMC4523465 25891780

[30] Kim JY Kim H Jeun SS Rha SJ Kim YH Ko YJ. Inhibition of NF-Kappa B Activation by Merlin. Biochem Biophys Res Commun (2002) 296(5):1295–302. doi: 10.1016/s0006-291x(02)02077-6 12207915

[31] Hong B Krusche CA Schwabe K Friedrich S Klein R Krauss JK. Cyclooxygenase-2 Supports Tumor Proliferation in Vestibular Schwannomas. Neurosurgery (2011) 68(4):1112–7. doi: 10.1227/NEU.0b013e318208f5c7 21221032

[32] Lewis D Roncaroli F Agushi E Mosses D Williams R Li KL. Inflammation and Vascular Permeability Correlate With Growth in Sporadic Vestibular Schwannoma. Neuro Oncol (2019) 21(3):314–25. doi: 10.1093/neuonc/noy177 PMC6380424 30388263

[33] de Vries WM Briaire-de Bruijn IH van Benthem PPG van der Mey AGL Hogendoorn PCW. M-CSF and IL-34 Expression as Indicators for Growth in Sporadic Vestibular Schwannoma. Virchows Arch (2019) 474(3):375–81. doi: 10.1007/s00428-018-2503-1 PMC6515692 30580386

[34] Kontorinis G Crowther JA Iliodromiti S Taylor WAS Locke R. Neutrophil to Lymphocyte Ratio as a Predictive Marker of Vestibular Schwannoma Growth. Otol Neurotol (2016) 37(5):580–5. doi: 10.1097/mao.0000000000001026 27093024

[35] Sass HCR Hansen M Borup R Nielsen FC Caye-Thomasen P. Tumor miRNA Expression Profile Is Related to Vestibular Schwannoma Growth Rate. Acta Neurochir (2020) 162(5):1187–95. doi: 10.1007/s00701-020-04238-4 32016588

[36] Cioffi JA Yue WY Mendolia-Loffredo S Hansen KR Wackym PA Hansen MR. MicroRNA-21 Overexpression Contributes to Vestibular Schwannoma Cell Proliferation and Survival. Otol Neurotol (2010) 31(9):1455–62. doi: 10.1097/MAO.0b013e3181f20655 PMC2978772 20856158

[37] Marioni G Nicol L Cazzador D Pavone C D'Avella D Martini A. Endoglin (CD105) Expression in Neurofibromatosis Type 2 Vestibular Schwannoma. Head Neck J Sci Spec (2019) 41(10):3612–7. doi: 10.1002/hed.25881 31313389

[38] Kramer F Stoever T Warnecke A Diensthuber M Lenarz T Wissel K. BDNF mRNA Expression Is Significantly Upregulated in Vestibular Schwannomas and Correlates With Proliferative Activity. J Neurooncol (2010) 98(1):31–9. doi: 10.1007/s11060-009-0063-6 19937367

[39] Xia L Yang S Wang CD Yu EX Zhang HL Zhang Y. Immunohistochemical Profiles of Matrix Metalloproteinases and Vascular Endothelial Growth Factor Overexpression in the Antoni B Area of Vestibular Schwannomas. World Neurosurg (2020) 144:E72–9. doi: 10.1016/j.wneu.2020.07.208 32758656

[40] Moller MN Werther K Nalla A Stangerup SE Thomsen J Bog-Hansen TC. Angiogenesis in Vestibular Schwannomas: Expression of Extracellular Matrix Factors MMP-2, MMP-9, and TIMP-1. Laryngoscope (2010) 120(4):657–62. doi: 10.1002/lary.20834 20205165

[41] Ren Y Hyakusoku H Sagers JE Landegger LD Welling DB Stankovic KM. MMP-14 (MT1-MMP) Is a Biomarker of Surgical Outcome and a Potential Mediator of Hearing Loss in Patients With Vestibular Schwannomas. Front Cell Neurosci (2020) 14:191. doi: 10.3389/fncel.2020.00191 32848608 PMC7424165

[42] Breun M Schwerdtfeger A Martellotta DD Kessler AF Monoranu CM Matthies C. ADAM9: A Novel Player in Vestibular Schwannoma Pathogenesis. Oncol Lett (2020) 19(3):1856–64. doi: 10.3892/ol.2020.11299 PMC7039135 32194680

[43] Nattmann A Breun M Monoranu CM Matthies C Ernestus RI Lohr M. Analysis of ADAM9 Regulation and Function in Vestibular Schwannoma Primary Cells. BMC Res Notes (2020) 13(1):528. doi: 10.1186/s13104-020-05378-7 33176868 PMC7659081

[44] Ariyannur PS Vikkath N Pillai AB. Cerebrospinal Fluid Hyaluronan and Neurofibromatosis Type 2. Cancer Microenviron (2018) 11(2-3):125–33. doi: 10.1007/s12307-018-0216-2 PMC6250613 30145722

[45] Huang X Xu J Shen YW Zhang L Xu M Chen MY. Protein Profiling of Cerebrospinal Fluid From Patients Undergoing Vestibular Schwannoma Surgery and Clinical Significance. BioMed Pharmacother (2019) 116:108985. doi: 10.1016/j.biopha.2019.108985 31146115

[46] Asthagiri AR Parry DM Butman JA Kim HJ Tsilou ET Zhuang Z. Neurofibromatosis Type 2. Lancet (2009) 373(9679):1974–86. doi: 10.1016/s0140-6736(09)60259-2 PMC4748851 19476995

[47] Lee JD Kwon TJ Kim UK Lee WS. Genetic and Epigenetic Alterations of the NF2 Gene in Sporadic Vestibular Schwannomas. PloS One (2012) 7(1):5. doi: 10.1371/journal.pone.0030418 PMC3266248 22295085

[48] Bretscher A Edwards K Fehon RG. ERM Proteins and Merlin: Integrators at the Cell Cortex. Nat Rev Mol Cell Biol (2002) 3(8):586–99. doi: 10.1038/nrm882 12154370

[49] Zoch A Mayerl S Schulz A Greither T Frappart L Rubsam J. Merlin Isoforms 1 and 2 Both Act as Tumour Suppressors and Are Required for Optimal Sperm Maturation. PloS One (2015) 10(8):25. doi: 10.1371/journal.pone.0129151 PMC4530865 26258444

[50] Maccollin M Ramesh V Jacoby LB Louis DN Rubio MP Pulaski K. Mutational Analysis of Patients With Neurofibromatosis-2. Am J Hum Genet (1994) 55(2):314–20. PMC1918355 7913580

[51] Goutagny S Raymond E Esposito-Farese M Trunet S Mawrin C Bernardeschi D. Phase II Study of Mtorc1 Inhibition by Everolimus in Neurofibromatosis Type 2 Patients With Growing Vestibular Schwannomas. J Neurooncol (2015) 122(2):313–20. doi: 10.1007/s11060-014-1710-0 25567352

[52] Petrilli AM Fuse MA Donnan MS Bott M Sparrow NA Tondera D. A Chemical Biology Approach Identified PI3K as a Potential Therapeutic Target for Neurofibromatosis Type 2. Am J Transl Res (2014) 6(5):471–93. PMC4212923 25360213

[53] Kaempchen K Mielke K Utermark T Langmesser S Hanemann CO. Upregulation of the Rac1/JNK Signaling Pathway in Primary Human Schwannoma Cells. Hum Mol Genet (2003) 12(11):1211–21. doi: 10.1093/hmg/ddg146 12761036

[54] Li W Cooper J Zhou L Yang C Erdjument-Bromage H Zagzag D. Merlin/NF2 Loss-Driven Tumorigenesis Linked to CRL4(DCAF1)-Mediated Inhibition of the Hippo Pathway Kinases Lats1 and 2 in the Nucleus. Cancer Cell (2014) 26(1):48–60. doi: 10.1016/j.ccr.2014.05.001 25026211 PMC4126592

[55] Mandati V Del Maestro L Dingli F Lombard B Loew D Molinie N. Phosphorylation of Merlin by Aurora A Kinase Appears Necessary for Mitotic Progression. J Biol Chem (2019) 294(35):12992–3005. doi: 10.1074/jbc.RA118.006937 PMC6721933 31296571

[56] McClatchey AI Fehon RG. Merlin and the ERM Proteins - Regulators of Receptor Distribution and Signaling at the Cell Cortex. Trends Cell Biol (2009) 19(5):198–206. doi: 10.1016/j.tcb.2009.02.006 19345106 PMC2796113

[57] Lallemand D Saint-Amaux AL Giovannini M. Tumor-Suppression Functions of Merlin Are Independent of Its Role as an Organizer of the Actin Cytoskeleton in Schwann Cells. J Cell Sci (2009) 122(22):4141–9. doi: 10.1242/jcs.045914 19910496

[58] Sher I Hanemann CO Karplus PA Bretscher A. The Tumor Suppressor Merlin Controls Growth in Its Open State, and Phosphorylation Converts It to a Less-Active More-Closed State. Dev Cell (2012) 22(4):703–5. doi: 10.1016/j.devcel.2012.03.008 PMC3725555 22516197

[59] Xing WC Li M Zhang FY Ma X Long JF Zhou H. The Conformation Change and Tumor Suppressor Role of Merlin Are Both Independent of Serine 518 Phosphorylation. Biochem Biophys Res Commun (2017) 493(1):46–51. doi: 10.1016/j.bbrc.2017.09.077 28919412

[60] Shaw RJ Paez JG Curto M Yaktine A Pruitt WM Saotome I. The Nf2 Tumor Suppressor, Merlin, Functions in Rac-Dependent Signaling. Dev Cell (2001) 1(1):63–72. doi: 10.1016/s1534-5807(01)00009-0 11703924

[61] Ye KQ. Phosphorylation of Merlin Regulates Its Stability and Tumor Suppressive Activity. Cell Adhes Migr (2007) 1(4):196–8. doi: 10.4161/cam.1.4.5192 PMC2634106 19262146

[62] Wickremesekera A Hovens CM Kaye AH. Expression of ErbB-1 and 2 in Vestibular Schwannomas. J Clin Neurosci (2007) 14(12):1199–206. doi: 10.1016/j.jocn.2007.05.009 17964790

[63] Altuna X Lopez JP Yu MA Arandazi MJ Harris JP Wang-Rodriguez J. Potential Role of Imatinib Mesylate (Gleevec, STI-571) in the Treatment of Vestibular Schwannoma. Otol Neurotol (2011) 32(1):163–70. doi: 10.1097/MAO.0b013e3182009665 21157293

[64] Taurone S Bianchi E Attanasio G Di Gioia C Ierino R Carubbi C. Immunohistochemical Profile of Cytokines and Growth Factors Expressed in Vestibular Schwannoma and in Normal Vestibular Nerve Tissue. Mol Med Rep (2015) 12(1):737–45. doi: 10.3892/mmr.2015.3415 25738867

[65] Ebrahem Q Chaurasia SS Vasanji A Qi JH Klenotic PA Cutler A. Cross-Talk Between Vascular Endothelial Growth Factor and Matrix Metalloproteinases in the Induction of Neovascularization *In Vivo* . Am J Pathol (2010) 176(1):496–503. doi: 10.2353/ajpath.2010.080642 19948826 PMC2797907

[66] Tamura R Tanaka T Akasaki Y Murayama Y Yoshida K Sasaki H. The Role of Vascular Endothelial Growth Factor in the Hypoxic and Immunosuppressive Tumor Microenvironment: Perspectives for Therapeutic Implications. Med Oncol (Northwood London England) (2019) 37(1):2. doi: 10.1007/s12032-019-1329-2 31713115

[67] Koutsimpelas D Bjelopavlovic M Yetis R Frauenknecht K Adryan B Schmidtmann I. The VEGF/VEGF-R Axis in Sporadic Vestibular Schwannomas Correlates With Irradiation and Disease Recurrence. Orl J Otorhinolaryngol Head Neck Surg (2012) 74(6):330–8. doi: 10.1159/000346238 23344215

[68] Alanin MC Klausen C Caye-Thomasen P Thomsen C Fugleholm K Poulsgaard L. The Effect of Bevacizumab on Vestibular Schwannoma Tumour Size and Hearing in Patients With Neurofibromatosis Type 2. Eur Arch Otorhinolaryngol (2015) 272(12):3627–33. doi: 10.1007/s00405-014-3398-3 25421643

[69] Gao X Zhao YC Stemmer-Rachamimov AO Liu H Huang PG Chin SM. Anti-VEGF Treatment Improves Neurological Function and Augments Radiation Response in NF2 Schwannoma Model. Proc Natl Acad Sci USA (2015) 112(47):14676–81. doi: 10.1073/pnas.1512570112 PMC4664377 26554010

[70] Yi D Kuo SZ Zheng H Abhold EL Brown CM Doherty JK. Activation of PDGFR and EGFR Promotes the Acquisition of a Stem Cell-Like Phenotype in Schwannomas. Otol Neurotol (2012) 33(9):1640–7. doi: 10.1097/MAO.0b013e31826a540d 22935817

[71] Fredriksson L Li H Eriksson U. The PDGF Family: Four Gene Products Form Five Dimeric Isoforms. Cytokine Growth Factor Rev (2004) 15(4):197–204. doi: 10.1016/j.cytogfr.2004.03.007 15207811

[72] Yener U Avsar T Akgun E Seker A Bayri Y Kilic T. Assessment of Antiangiogenic Effect of Imatinib Mesylate on Vestibular Schwannoma Tumors Using *In Vivo* Corneal Angiogenesis Assay Laboratory Investigation. J Neurosurg (2012) 117(4):697–704. doi: 10.3171/2012.6.Jns112263 22900848

[73] Thomas KA Gimenezgallego G. Fibroblast Growth-Factors - Broad-Spectrum Mitogens With Potent Angiogenic Activity. Trends Biochem Sci (1986) 11(2):81–4. doi: 10.1016/0968-0004(86)90271-9

[74] Labanca E Vazquez ES Corn PG Roberts JM Wang F Logothetis CJ. Fibroblast Growth Factors Signaling in Bone Metastasis. Endocr Relat Cancer (2020) 27(7):R255–65. doi: 10.1530/erc-19-0472 PMC7274538 32369771

[75] Dilwali S Lysaght A Roberts D Barker FG McKenna MJ Stankovic KM. Sporadic Vestibular Schwannomas Associated With Good Hearing Secrete Higher Levels of Fibroblast Growth Factor 2 Than Those Associated With Poor Hearing Irrespective of Tumor Size. Otol Neurotol (2013) 34(4):748–54. doi: 10.1097/MAO.0b013e31828048ec PMC3655133 23512073

[76] Plotkin SR Duda DG Muzikansky A Allen J Blakeley J Rosser T. Multicenter, Prospective, Phase II and Biomarker Study of High-Dose Bevacizumab as Induction Therapy in Patients With Neurofibromatosis Type 2 and Progressive Vestibular Schwannoma. J Clin Oncol (2019) 37(35):3446–54. doi: 10.1200/jco.19.01367 PMC7098833 31626572

[77] Weerda HG Gamberger TI Siegner A Gjuric M Tamm ER. Effects of Transforming Growth Factor- ß 1 and Basic Fibroblast Growth Factor on Proliferation of Cell Cultures Derived From Human Vestibular Nerve Schwannoma. Acta Otolaryngol (1998) 118(3):337–43. doi: 10.1080/00016489850183412 9655207

[78] Ahmad ZK Brown CM Cueva RA Ryan AF Doherty JK. ErbB Expression, Activation, and Inhibition With Lapatinib and Tyrphostin (AG825) in Human Vestibular Schwannomas. Otol Neurotol (2011) 32(5):841–7. doi: 10.1097/MAO.0b013e31821f7d88 PMC3584631 21659924

[79] Bush ML Burns SS Oblinger J Davletova S Chang L-S Welling DB. Treatment of Vestibular Schwannoma Cells With ErbB Inhibitors. Otol Neurotol (2012) 33(2):244–57. doi: 10.1097/MAO.0b013e31823e287f PMC3522123 22222570

[80] Clark JJ Provenzano M Diggelmann HR Xu N Hansen SS Hansen MR. The ErbB Inhibitors Trastuzumab and Erlotinib Inhibit Growth of Vestibular Schwannoma Xenografts in Nude Mice: A Preliminary Study. Otol Neurotol (2008) 29(6):846–53. doi: 10.1097/MAO.0b013e31817f7398 PMC2652856 18636037

[81] Fuse MA Dinh CT Vitte J Kirkpatrick J Mindos T Plati SK. Preclinical Assessment of MEK1/2 Inhibitors for Neurofibromatosis Type 2-Associated Schwannomas Reveals Differences in Efficacy and Drug Resistance Development. Neuro Oncol (2019) 21(4):486–97. doi: 10.1093/neuonc/noz002 PMC6422635 30615146

[82] Zinatizadeh MR Momeni SA Zarandi PK Chalbatani GM Dana H Mirzaei HR. The Role and Function of Ras-Association Domain Family in Cancer: A Review. Genes Dis (2019) 6(4):378–84. doi: 10.1016/j.gendis.2019.07.008 PMC6889020 31832517

[83] Agnihotri S Gugel I Remke M Bornemann A Pantazis G Mack SC. Gene-Expression Profiling Elucidates Molecular Signaling Networks That Can Be Therapeutically Targeted in Vestibular Schwannoma. J Neurosurg (2014) 121(6):1434–45. doi: 10.3171/2014.6.jns131433 25245477

[84] Slack-Davis JK Eblen ST Zecevic M Boerner SA Tarcsafalvi A Diaz HB. PAKI Phosphorylation of MEK1 Regulates Fibronectin-Stimulated MAPK Activation. J Cell Biol (2003) 162(2):281–91. doi: 10.1083/jcb.200212141 PMC2172784 12876277

[85] Li WQ Chong HR Guan KL. Function of the Rho Family GTPases in Ras-Stimulated Raf Activation. J Biol Chem (2001) 276(37):34728–37. doi: 10.1074/jbc.M103496200 11457831

[86] Morrison H Sperka T Manent J Giovannini M Ponta H Herrlich P. Merlin/neurofibromatosis Type 2 Suppresses Growth by Inhibiting the Activation of Ras and Rac. Cancer Res (2007) 67(2):520–7. doi: 10.1158/0008-5472.Can-06-1608 17234759

[87] Flaiz C Chernoff J Ammoun S Peterson JR Hanemann CO. PAK Kinase Regulates Rac GTPase and Is a Potential Target in Human Schwannomas. Exp Neurol (2009) 218(1):137–44. doi: 10.1016/j.expneurol.2009.04.019 PMC2760977 19409384

[88] Mercado-Pimentel ME Miller C Rolph DN Villalobos EF Dunn AM Mohan PM. Inhibiting P21-Activated Kinase Induces Cell Death in Vestibular Schwannoma and Meningioma *via* Mitotic Catastrophe. Otol Neurotol (2017) 38(1):139–46. doi: 10.1097/mao.0000000000001247 PMC5154791 27755359

[89] Yang PZ Sun DZ Jiang F. Ailanthone Promotes Human Vestibular Schwannoma Cell Apoptosis and Autophagy by Downregulation of miR-21. Oncol Res (2018) 26(6):941–8. doi: 10.3727/096504018x15149775533331 PMC7844645 29298734

[90] Riecken LB Zoch A Wiehl U Reichert S Scholl I Cui Y. CPI-17 Drives Oncogenic Ras Signaling in Human Melanomas *via* Ezrin-Radixin-Moesin Family Proteins. Oncotarget (2016) 7(48):78242–54. doi: 10.18632/oncotarget.12919 PMC5346635 27793041

[91] Cui Y Groth S Troutman S Carlstedt A Sperka T Riecken LB. The NF2 Tumor Suppressor Merlin Interacts With Ras and RasGAP, Which may Modulate Ras Signaling. Oncogene (2019) 38(36):6370–81. doi: 10.1038/s41388-019-0883-6 PMC6756068 31312020

[92] Sperka T Geissler KJ Merkel U Scholl I Rubio I Herrlich P. Activation of Ras Requires the ERM-Dependent Link of Actin to the Plasma Membrane. PloS One (2011) 6(11):14. doi: 10.1371/journal.pone.0027511 PMC3221661 22132106

[93] Li Y Zhou H Li F Chan SW Lin Z Wei Z. Angiomotin Binding-Induced Activation of Merlin/NF2 in the Hippo Pathway. Cell Res (2015) 25(7):801–17. doi: 10.1038/cr.2015.69 PMC4493278 26045165

[94] Reginensi A Enderle L Gregorieff A Johnson RL Wrana JL McNeill H. A Critical Role for NF2 and the Hippo Pathway in Branching Morphogenesis. Nat Commun (2016) 7:12309. doi: 10.1038/ncomms12309 27480037 PMC4974664

[95] Boin A Couvelard A Couderc C Brito I Filipescu D Kalamarides M. Proteomic Screening Identifies a YAP-Driven Signaling Network Linked to Tumor Cell Proliferation in Human Schwannomas. Neuro Oncol (2014) 16(9):1196–209. doi: 10.1093/neuonc/nou020 PMC4136892 24558021

[96] Cooper J Xu Q Zhou L Pavlovic M Ojeda V Moulick K. Combined Inhibition of NEDD8-Activating Enzyme and mTOR Suppresses NF2 Loss-Driven Tumorigenesis. Mol Cancer Ther (2017) 16(8):1693–704. doi: 10.1158/1535-7163.mct-16-0821 PMC5929164 28468780

[97] Jiang XJ Wang J Deng XY Xiong F Zhang SS Gong ZJ. The Role of Microenvironment in Tumor Angiogenesis. J Exp Clin Cancer Res (2020) 39(1):19. doi: 10.1186/s13046-020-01709-5 32993787 PMC7526376

[98] Wei F Wang D Wei JY Tang NW Tang L Xiong F. Metabolic Crosstalk in the Tumor Microenvironment Regulates Antitumor Immunosuppression and Immunotherapy Resisitance. Cell Mol Life Sci (2021) 78(1):173–93. doi: 10.1007/s00018-020-03581-0 PMC11072448 32654036

[99] Lai S Huang L Luo S Liu Z Dong J Wang L. Systemic Inflammatory Indices Predict Tumor Response to Neoadjuvant Chemoradiotherapy for Locally Advanced Rectal Cancer. Oncol Lett (2020) 20(3):2763–70. doi: 10.3892/ol.2020.11812 PMC7400706 32782593

[100] Sawada R Akiyoshi T Kitagawa Y Hiyoshi Y Mukai T Nagasaki T. Systemic Inflammatory Markers Combined With Tumor-Infiltrating Lymphocyte Density for the Improved Prediction of Response to Neoadjuvant Chemoradiotherapy in Rectal Cancer. Ann Surg Oncol (2021). doi: 10.1245/s10434-021-09975-z. in press. 33876358

[101] de Vries M Hogendoorn PCW Briaire-de Bruyn I Malessy MJA van der Mey AGL. Intratumoral Hemorrhage, Vessel Density, and the Inflammatory Reaction Contribute to Volume Increase of Sporadic Vestibular Schwannomas. Virchows Arch (2012) 460(6):629–36. doi: 10.1007/s00428-012-1236-9 PMC3371334 22555941

[102] Han JH Baek KH Lee YW Hur YK Kim HJ Moon IS. Comparison of Clinical Characteristics and Surgical Outcomes of Cystic and Solid Vestibular Schwannomas. Otol Neurotol (2018) 39(5):E381–6. doi: 10.1097/mao.0000000000001813 29738391

[103] Motolani A Martin M Sun MY Lu T. Phosphorylation of the Regulators, a Complex Facet of NF-Kappa B Signaling in Cancer. Biomolecules (2021) 11(1):13. doi: 10.3390/biom11010015 PMC7823564 33375283

[104] Pereira SG Oakley F. Nuclear Factor-Kappa B1: Regulation and Function. Int J Biochem Cell Biol (2008) 40(8):1425–30. doi: 10.1016/j.biocel.2007.05.004 17693123

[105] Karin M Delhase M. The I Kappa B Kinase (IKK) and NF-Kappa B: Key Elements of Proinflammatory Signalling. Semin Immunol (2000) 12(1):85–98. doi: 10.1006/smim.2000.0210 10723801

[106] Sun SC. The Noncanonical NF-Kappa B Pathway. Immunol Rev (2012) 246:125–40. doi: 10.1111/j.1600-065X.2011.01088.x PMC3313452 22435551

[107] Hoesel B Schmid JA. The Complexity of NF-κb Signaling in Inflammation and Cancer. Mol Cancer (2013) 12(1):86. doi: 10.1186/1476-4598-12-86 23915189 PMC3750319

[108] Gehlhausen JR Hawley E Wahle BM He YZ Edwards D Rhodes SD. A Proteasome-Resistant Fragment of NIK Mediates Oncogenic NF-Kappa B Signaling in Schwannomas. Hum Mol Genet (2019) 28(4):572–83. doi: 10.1093/hmg/ddy361 PMC6489415 30335132

[109] Ahmad I Yue WY Fernando A Clark JJ Woodson EA Hansen MR. P75(NTR) Is Highly Expressed in Vestibular Schwannomas and Promotes Cell Survival by Activating Nuclear Transcription Factor Kappa B. Glia (2014) 62(10):1699–712. doi: 10.1002/glia.22709 PMC4150679 24976126

[110] Behling F Ries V Skardelly M Gepfner-Tuma I Schuhmann M Ebner FH. COX2 Expression Is Associated With Proliferation and Tumor Extension in Vestibular Schwannoma But Is Not Influenced by Acetylsalicylic Acid Intake. Acta Neuropathol Commun (2019) 7:105. doi: 10.1186/s40478-019-0760-0 31291992 PMC6621994

[111] Xu J Zhang Y Shi Y Yin D Dai P Zhao W. Identification of Predictive Proteins and Biological Pathways for the Tumorigenicity of Vestibular Schwannoma by Proteomic Profiling. Proteomics Clin Appl (2019) 13(5):1800175. doi: 10.1002/prca.201800175 31120176

[112] Gong ZX Huang WG Wang BY Liang N Long SK Li WJ. Interplay Between Cyclooxygenase-2 and microRNAs in Cancer. Mol Med Rep (2021) 23(5):1–10. doi: 10.3892/mmr.2021.11986 PMC7974460 33760116

[113] Davila-Gonzalez D Chang JC Billiar TR. NO and COX2: Dual Targeting for Aggressive Cancers. Proc Natl Acad Sci USA (2017) 114(52):13591–3. doi: 10.1073/pnas.1717440114 PMC5748226 29237749

[114] Goradel NH Najafi M Salehi E Farhood B Mortezaee K. Cyclooxygenase-2 in Cancer: A Review. J Cell Physiol (2019) 234(5):5683–99. doi: 10.1002/jcp.27411 30341914

[115] Montezuma MAP Fonseca FP Benites BM Soares CD do Amaral-Silva GK de Almeida OP. COX-2 as a Determinant of Lower Disease-Free Survival for Patients Affected by Ameloblastoma. Pathol Res Pract (2018) 214(6):907–13. doi: 10.1016/j.prp.2018.03.014 29559247

[116] Ma J Cai ZL Wei HL Liu XL Zhao QL Zhang T. The Anti-Tumor Effect of Aspirin: What We Know and What We Expect. BioMed Pharmacother (2017) 95:656–61. doi: 10.1016/j.biopha.2017.08.085 28881293

[117] Wang P Shen YP Zhao L. Chitosan Nanoparticles Loaded With Aspirin and 5-Fluororacil Enable Synergistic Antitumour Activity Through the Modulation of NF-Kappa B/COX-2 Signalling Pathway. IET Nanobiotechnol (2020) 14(6):479–84. doi: 10.1049/iet-nbt.2020.0002 PMC8676317 32755957

[118] Jiang W Yan Y Chen MY Luo GY Hao JJ Pan JJ. Aspirin Enhances the Sensitivity of Colon Cancer Cells to Cisplatin by Abrogating the Binding of NF-Kappa B to the COX-2 Promoter. Aging-US (2020) 12(1):611–27. doi: 10.18632/aging.102644 PMC6977689 31905343

[119] Kandathil CK Dilwali S Wu CC Ibrahimov M McKenna MJ Lee H. Aspirin Intake Correlates With Halted Growth of Sporadic Vestibular Schwannoma *In Vivo* . Otol Neurotol (2014) 35(2):353–7. doi: 10.1097/mao.0000000000000189 24448296

[120] Kandathil CK Cunnane ME McKenna MJ Curtin HD Stankovic KM. Correlation Between Aspirin Intake and Reduced Growth of Human Vestibular Schwannoma: Volumetric Analysis. Otol Neurotol (2016) 37(9):1428–34. doi: 10.1097/mao.0000000000001180 27631829

[121] Ignacio KHD Espiritu AI Diestro JDB Chan KI Dmytriw AA Omar AT. Efficacy of Aspirin for Sporadic Vestibular Schwannoma: A Meta-Analysis. Neurol Sci (2021). doi: 10.1007/s10072-021-05193-3. in press. 33772351

[122] Mehraj U Qayoom H Mir MA. Prognostic Significance and Targeting Tumor-Associated Macrophages in Cancer: New Insights and Future Perspectives. Breast Cancer (2021) 28(3):1–17. doi: 10.1007/s12282-021-01231-2 33661479

[123] Huang YC Feng ZP. The Good and Bad of Microglia/Macrophages: New Hope in Stroke Therapeutics. Acta Pharmacol Sin (2013) 34(1):6–7. doi: 10.1038/aps.2012.178 23262666 PMC4086493

[124] Rogers TL Holen I. Tumour Macrophages as Potential Targets of Bisphosphonates. J Trans Med (2011) 9:17. doi: 10.1186/1479-5876-9-177 PMC3215187 22005011

[125] Cao LL Che XF Qiu XS Li Z Yang BW Wang S. M2 Macrophage Infiltration Into Tumor Islets Leads to Poor Prognosis in Non-Small-Cell Lung Cancer. Cancer Manage Res (2019) 11:6125–38. doi: 10.2147/cmar.S199832 PMC6613613 31308749

[126] Hu H Tu WZ Chen YG Zhu M Jin H Huang T. The Combination of PKM2 Overexpression and M2 Macrophages Infiltration Confers a Poor Prognosis for PDAC Patients. J Cancer (2020) 11(8):2022–31. doi: 10.7150/jca.38981 PMC7052945 32127930

[127] Lewis D Donofrio CA O'Leary C Li K-L Zhu X Williams R. The Microenvironment in Sporadic and Neurofibromatosis Type II-Related Vestibular Schwannoma: The Same Tumor or Different? A Comparative Imaging and Neuropathology Study. J Neurosurg (2021) 134(5):1419–29. doi: 10.3171/2020.3.Jns193230 32470937

[128] Bi WL Gupta S Mei Y Al Abdulmohsen S Larsen AG Unadkat P. Immunophenotype of Vestibular Schwannomas. Otol Neurotol (2020) 41(10):E1290–E6. doi: 10.1097/mao.0000000000002782 33492804

[129] Chow MT Moeller A Smyth MJ. Inflammation and Immune Surveillance in Cancer. Semin Cancer Biol (2012) 22(1):23–32. doi: 10.1016/j.semcancer.2011.12.004 22210181

[130] Templeton AJ McNamara MG Seruga B Vera-Badillo FE Aneja P Ocana A. Prognostic Role of Neutrophil-To-Lymphocyte Ratio in Solid Tumors: A Systematic Review and Meta-Analysis. J Natl Cancer Inst (2014) 106(6):dju124. doi: 10.1093/jnci/dju124 24875653

[131] Tikka T Yiannakis CP Stapleton E Locke R Crowther JA Taylor WAS. Spontaneous Vestibular Schwannoma Regression: A Case-Control Study. Otol Neurotol (2018) 39(10):E1118–24. doi: 10.1097/mao.0000000000001962 30106843

[132] Ying SY Lin SL. Current Perspectives in Intronic Micro RNAs (miRNAs). J BioMed Sci (2006) 13(1):5–15. doi: 10.1007/s11373-005-9036-8 16228283

[133] Lei YH Guo P Li XG Zhang YY Du T. Identification of Differentially Expressed miRNAs and mRNAs in Vestibular Schwannoma by Integrated Analysis. BioMed Res Int (2019) 2019:10. doi: 10.1155/2019/7267816 PMC6594327 31309113

[134] Saydam O Senol O Wurdinger T Mizrak A Ozdener GB Stemmer-Rachamimov AO. miRNA-7 Attenuation in Schwannoma Tumors Stimulates Growth by Upregulating Three Oncogenic Signaling Pathways. Cancer Res (2011) 71(3):852–61. doi: 10.1158/0008-5472.Can-10-1219 PMC3072568 21156648

[135] Zhao X Hou YX Tuo ZZ Wei FM. Application Values of miR-194 and miR-29 in the Diagnosis and Prognosis of Gastric Cancer. Exp Ther Med (2018) 15(5):4179–84. doi: 10.3892/etm.2018.5931 PMC5920402 29725366

[136] Liu QL Geng PS Shi LY Wang Q Wang PL. miR-29 Promotes Osteosarcoma Cell Proliferation and Migration by Targeting PTEN. Oncol Lett (2019) 17(1):883–90. doi: 10.3892/ol.2018.9646 PMC6313002 30655843

[137] Jiang HS Zhang G Wu JH Jiang CP. Diverse Roles of miR-29 in Cancer (Review). Oncol Rep (2014) 31(4):1509–16. doi: 10.3892/or.2014.3036 24573597

[138] Strong AMP Setaluri V Spiegelman VS. microRNA-340 as a Modulator of RAS-RAF-MAPK Signaling in Melanoma. Arch Biochem Biophys (2014) 563:118–24. doi: 10.1016/j.abb.2014.07.012 PMC4221550 25043973

[139] Rong JP Xu L Hu YY Liu F Yu YR Guo HY. Inhibition of Let-7b-5p Contributes to an Anti-Tumorigenic Macrophage Phenotype Through the SOCS1/STAT Pathway in Prostate Cancer. Cancer Cell Int (2020) 20(1):15. doi: 10.1186/s12935-020-01563-7 33005103 PMC7526222

[140] Peng H Yang H Xiang X Li SG. MicroRNA-221 Participates in Cerebral Ischemic Stroke by Modulating Endothelial Cell Function by Regulating the PTEN/PI3K/AKT Pathway. Exp Ther Med (2020) 19(1):443–50. doi: 10.3892/etm.2019.8263 PMC6913279 31885694

[141] Sucheta Kataria SP Malik S Yadav R Kapil R Sen R. Histomorphological and Morphometric Evaluation of Microvessel Density in Nodal Non-Hodgkin Lymphoma Using CD34 and CD105. J Lab Phys (2021) 22(9):7. doi: 10.1055/s-0041-1726569 PMC8205556 34149231

[142] Burghardt I Ventura E Weiss T Schroeder JJ Seystahl K Zielasek C. Endoglin and TGF-Beta Signaling in Glioblastoma. Cell Tissue Res (2021) 384(3):613–24. doi: 10.1007/s00441-020-03323-5 PMC8211614 33471197

[143] Mo FZ Duan SL Jiang XB Yang XM Hou XQ Shi W. Nanobody-Based Chimeric Antigen Receptor T Cells Designed by CRISPR/Cas9 Technology for Solid Tumor Immunotherapy. Signal Transduct Target Ther (2021) 6(1):12. doi: 10.1038/s41392-021-00462-1 33627635 PMC7904846

[144] Liu YM Paauwe M Nixon AB Hawinkels L. Endoglin Targeting: Lessons Learned and Questions That Remain. Int J Mol Sci (2021) 22(1):15. doi: 10.3390/ijms22010147 PMC7795616 33375670

[145] de Vries I Schmitt H Lenarz T Prenzler N Alvi S Staecker H. Detection of BDNF-Related Proteins in Human Perilymph in Patients With Hearing Loss. Front Neurosci (2019) 13:214. doi: 10.3389/fnins.2019.00214 30971872 PMC6445295

[146] Fields GB. Mechanisms of Action of Novel Drugs Targeting Angiogenesis-Promoting Matrix Metalloproteinases. Front Immunol (2019) 10:1278. doi: 10.3389/fimmu.2019.01278 31214203 PMC6558196

[147] Xu JH Ma J Shi YX Yin DM Zhang Y Dai PD. Differential Protein Expression Between Cystic and Solid Vestibular Schwannoma Using Tandem Mass Tag-Based Quantitative Proteomic Analysis. Proteomics Clin Appl (2020) 14(4):1900112. doi: 10.1002/prca.201900112 32157794

[148] Moon KS Jung S Seo SK Jung TY Kim IY Ryu HH. Cystic Vestibular Schwannomas: A Possible Role of Matrix Metalloproteinase-2 in Cyst Development and Unfavorable Surgical Outcome. J Neurosurg (2007) 106(5):866–71. doi: 10.3171/jns.2007.106.5.866 17542531

[149] Oria VO Lopatta P Schilling O. The Pleiotropic Roles of ADAM9 in the Biology of Solid Tumors. Cell Mol Life Sci (2018) 75(13):2291–301. doi: 10.1007/s00018-018-2796-x PMC11105608 29550974

[150] Mygind KJ Schwarz J Sahgal P Ivaska J Kveiborg M. Loss of ADAM9 Expression Impairs Beta 1 Integrin Endocytosis, Focal Adhesion Formation and Cancer Cell Migration. J Cell Sci (2018) 131(1):jcs205393. doi: 10.1242/jcs.205393 29142101

[151] Mygind KJ Storiko T Freiberg ML Samsoe-Petersen J Schwarz J Andersen OM. Sorting Nexin 9 (SNX9) Regulates Levels of the Transmembrane ADAM9 at the Cell Surface. J Biol Chem (2018) 293(21):8077–88. doi: 10.1074/jbc.RA117.001077 PMC5971451 29622675

[152] Chang L Gong FC Cai HF Li ZH Cui YB. Combined RNAi Targeting Human Stat3 and ADAM9 as Gene Therapy for Non-Small Cell Lung Cancer. Oncol Lett (2016) 11(2):1242–50. doi: 10.3892/ol.2015.4018 PMC4734062 26893726

[153] Chen WL Lu Q Li SY Zhang XY Xue XH. microRNA-1298 Inhibits the Malignant Behaviors of Breast Cancer Cells via Targeting ADAM9. Biosci Rep (2020) 40:12. doi: 10.1042/bsr20201215 PMC7729294 33146718

[154] Gol MAK Lund TC Levine SC Adams ME. Quantitative Proteomics of Vestibular Schwannoma Cerebrospinal Fluid: A Pilot Study. Otolaryngol Head Neck Surg (2016) 154(5):902–6. doi: 10.1177/0194599816630544 26932958

[155] Xiao F Lv SG Zong ZT Wu L Tang XP Kuang W. Cerebrospinal Fluid Biomarkers for Brain Tumor Detection: Clinical Roles and Current Progress. Am J Transl Res (2020) 12(4):1379–96. PMC7191171 32355549

[156] Mattox AK Yan H Bettegowda C. The Potential of Cerebrospinal Fluid-Based Liquid Biopsy Approaches in CNS Tumors. Neuro Oncol (2019) 21(12):1509–18. doi: 10.1093/neuonc/noz156 PMC6917400 31595305

[157] Bai Y Liu YJ Wang H Xu Y Stamenkovic I Yu Q. Inhibition of the Hyaluronan-CD44 Interaction by Merlin Contributes to the Tumor-Suppressor Activity of Merlin. Oncogene (2007) 26(6):836–50. doi: 10.1038/sj.onc.1209849 16953231

[158] de Vries M Briaire-de Bruijn I Malessy MJ de Bruïne SF van der Mey AG Hogendoorn PC. Tumor-Associated Macrophages Are Related to Volumetric Growth of Vestibular Schwannomas. Otol Neurotol (2013) 34(2):347–52. doi: 10.1097/MAO.0b013e31827c9fbf 23295727

[159] Neff BA Voss SG Allen C Schroeder MA Driscoll CLW Link MJ. Bioluminescent Imaging of Intracranial Vestibular Schwannoma Xenografts in NOD/SCID Mice. Otol Neurotol (2009) 30(1):105–11. doi: 10.1097/MAO.0b013e31818b6cea PMC3918230 18931645

[160] Bonne N-X Vitte J Chareyre F Karapetyan G Khankaldyyan V Tanaka K. An Allograft Mouse Model for the Study of Hearing Loss Secondary to Vestibular Schwannoma Growth. J Neurooncol (2016) 129(1):47–56. doi: 10.1007/s11060-016-2150-9 27177628

[161] Szczupak M Pena SA Bracho O Mei C Bas E Fernandez-Valle C. Fluorescent Detection of Vestibular Schwannoma Using Intravenous Sodium Fluorescein *In Vivo* . Otol Neurotol (2021) 42(4):E503–11. doi: 10.1097/mao.0000000000002988 PMC8590806 33492057

[162] Helbing DL Schulz A Morrison H. Pathomechanisms in Schwannoma Development and Progression. Oncogene (2020) 39(32):5421–9. doi: 10.1038/s41388-020-1374-5 PMC7410823 32616891

[163] Lee HJ Yang YJ Jeong S Lee JD Choi SY Jung DW. Development of a Vestibular Schwannoma Xenograft Zebrafish Model for *In Vivo* Antitumor Drug Screening. Laryngoscope (2016) 126(12):E409–15. doi: 10.1002/lary.26043 27242319

[164] Breun M Martellotta DD Leberle A Nietzer S Baur F Ernestus R-I. 3D *In Vitro* Test System for Vestibular Schwannoma. J Neurosci Methods (2020) 336:108633. doi: 10.1016/j.jneumeth.2020.108633 32061689

[165] Wu L Vasilijic S Sun Y Chen J Landegger LD Zhang Y. Losartan Prevents Tumor-Induced Hearing Loss and Augments Radiation Efficacy in NF2 Schwannoma Rodent Models. Sci Transl Med (2021) 13(602):eabd4816. doi: 10.1126/scitranslmed.abd4816 34261799 PMC8409338

[166] Meyer HJ Leifels L Hamerla G Hohn AK Surov A. Histogram Analysis Parameters Derived From Conventional T1-And T2-Weighted Images Can Predict Different Histopathological Features Including Expression of Ki67, EGFR, VEGF, HIF-1 Alpha, and P53 and Cell Count in Head and Neck Squamous Cell Carcinoma. Mol Imag Biol (2019) 21(4):740–6. doi: 10.1007/s11307-018-1283-y 30284155

[167] Shcherbakova DM Baloban M Emelyanov AV Brenowitz M Guo P Verkhusha VV. Bright Monomeric Near-Infrared Fluorescent Proteins as Tags and Biosensors for Multiscale Imaging. Nat Commun (2016) 7:12405. doi: 10.1038/ncomms12405 27539380 PMC4992171

[168] Hessler M Jalilian E Xu Q Reddy S Horton L Elkin K. Melanoma Biomarkers and Their Potential Application for *In Vivo* Diagnostic Imaging Modalities. Int J Mol Sci (2020) 21(24):9583. doi: 10.3390/ijms21249583 PMC7765677 33339193

[169] Sewda K Coppola D Enkemann S Yue B Kim J Lopez AS. Cell-Surface Markers for Colon Adenoma and Adenocarcinoma. Oncotarget (2016) 7(14):17773–89. doi: 10.18632/oncotarget.7402 PMC4951249 26894861

[170] Morrison DR Sorace AG Hamilton E Moore LS Houson HA Udayakumar N. Predicting Schwannoma Growth in a Tumor Model Using Targeted Imaging. Otol Neurotol (2021) 42(5):e615–23. doi: 10.1097/MAO.0000000000003063 PMC9762121 33661237

